# A Novel Self‐Regulated, Non‐Directional Magnetic Thermos‐Brachytherapy ^125^I Seed Enhances Anticancer Efficacy by Rescuing Immune Escape

**DOI:** 10.1002/advs.202508091

**Published:** 2025-08-29

**Authors:** Bin Xu, Ye‐Ming Wu, Yi‐Fei Cheng, Lei Dong, Shuai‐Wen Ding, Sheng‐Rong Chen, Bin‐Da Chen, Ren‐Peng Yang, Wei‐Hua Zhang, Ya‐Jie Wang, Wei‐Pu Mao, Fan Shi, Zhi‐Cheng Jin, Jian‐Jian Chen, En‐Qi Qiao, Yu‐Qing Wang, Jin‐Ze Li, Peng Zeng, Cheng‐Wei Wu, Wei Zhang, Jin‐Bing Xie, Jian Lu, Gao‐Jun Teng

**Affiliations:** ^1^ Department of Urology Zhongda Hospital Medical School Southeast University Nanjing 210009 China; ^2^ Center of Interventional Radiology and Vascular Surgery Department of Radiology Zhongda Hospital Medical School Southeast University Nanjing 210009 China; ^3^ Basic Medicine Research and Innovation Center of Ministry of Education Zhongda Hospital National Innovation Platform for Integration of Medical Engineering Education (NMEE) (Southeast University) State Key Laboratory of Digital Medical Engineering Southeast University Nanjing 210009 China; ^4^ Department of Oncology Zhongda Hospital Medical School Southeast University Nanjing 210009 China; ^5^ State Key Laboratory of Structural Analysis Optimization and CAE Software for Industrial Equipment Department of Engineering Mechanics Dalian University of Technology Dalian 116024 China; ^6^ JACO PHARMACEUTICALS CO., LTD NingBo 315800 China; ^7^ Jiangsu Key Laboratory of Intelligent Medical Image Computing School of Future Technology Nanjing University of Information Science and Technology Nanjing 210044 China

**Keywords:** brachytherapy, composite seed, immune escape, mild hyperthermia, neutrophil

## Abstract

Integrating mild hyperthermia (MH) with ^125^I brachytherapy holds potential for overcoming treatment resistance and improving anticancer efficacy. Here, magnetic nanoparticles (MNPs) with a suitable Curie temperature are constructed and incorporated with silver rods coated with ^125^I to form composite seeds. In vitro simulations and in vivo validations demonstrated their effective performance in radiation dose and temperature control. Compared with traditional thermoseeds and previously reported MNPs, this composite seed exhibits direction‐independent and self‐regulated heating efficiency. Additionally, the titanium shell prevented MNPs leakage and enabled its repeated hyperthermia treatment capacity. Subsequently, the enhanced pancancer anticancer efficacy of the composite seed‐relied ^125^I@MH therapy is confirmed through cellular and animal experiments involving liver cancer and prostate cancer. Further tumor microenvironment investigations based on a subcutaneous liver cancer mouse model identified that ^125^I therapy recruited Cd274/Pd‐l1^+^ neutrophils and induced T‐cell exhaustion, leading to immune evasion and brachytherapy resistance. The addition of MH significantly reversed this effect, restoring the function of effector T cells (IFN‐γ^+^ T cells) and activating T‐cell immunity. In conclusion, this study developed a novel composite seed with superior anticancer efficacy, which holds promising therapeutic potential for the treatment of malignancies, particularly solid tumors, in future clinical practice.

## Graphical abstract

This work constructs magnetic nanoparticles (MNPs) and incorporates them with silver rods coated with ^125^I to form a novel composite seed, which exhibits direction‐independent and self‐regulated heating efficiency, as well as the capacity of preventing MNPs leakage and enabling repeated hyperthermia. Mechanistically, the composite seed‐relied combination therapy enhances anti‐cancer efficacy through rescuing PD‐L1^+^ neutrophil‐mediated immune escape.

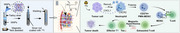

## 1 Introduction

Radiotherapy, including external beam radiotherapy (EBRT), proton therapy, and brachytherapy, is crucial for treating malignant tumors. In comparison, brachytherapy delivers radiation internally, with the radiation directed precisely to the target area without passing through healthy tissues.^[^ ^1^ ^]^ Iodine‐125 (^125^I) is a widely used radioactive source for permanent interstitial brachytherapy for various malignancies, including prostate cancer (PCa), head and neck tumors, and liver cancer.^[^ ^2^ ^]^

However, due to various factors, such as tumor microenvironment (TME) remodeling (including the induction of tissue hypoxia and immune evasion), tumors can develop resistance to radiotherapy, thereby limiting therapeutic efficacy.^[^ ^3^, ^4^, ^5^ ^]^ Thus, numerous studies have investigated potential combination therapeutic strategies, such as chemotherapy and immunotherapy, to enhance the treatment efficacy of ^125^I brachytherapy.^[^ ^4^, ^6^, ^7^, ^8^ ^]^ Among them, mild hyperthermia (MH) has emerged as a promising strategy. MH is a technique that raises the body or local tissue temperature to 39–45 °C and has been widely applied in cancer therapy.^[^ ^9^ ^]^ MH can relieve the hypoxic tumor microenvironment by promoting vasodilation,^[^ ^10^ ^]^ thereby increasing sensitivity to radiotherapy. Additionally, combined treatment approaches can reduce radiation doses, minimizing damage to normal tissues while ensuring therapeutic efficacy.^[^ ^9^ ^]^

Currently, MH technologies have evolved from traditional hot water baths to more precise, targeted heating methods using ultrasound, microwaves, radiofrequency, and nanotechnology.^[^ ^11^, ^12^ ^]^ However, when applied to tumors, these technologies face significant challenges. For example, it is difficult to precisely control the temperature in the treatment area, especially for deep‐seated tumors, making it challenging to reach the therapeutic temperature.^[^ ^13^, ^14^ ^]^ Additionally, MH alone is typically insufficient to eliminate all cancer cells, requiring combination with other therapies^[^ ^15^ ^]^; individual heterogeneity, including body mass index and ethnicity, significantly affects the efficacy of hyperthermia, making it challenging to achieve standardization.^[^ ^16^ ^]^ Moreover, MH has low specificity for tumors, which increases the risk of damage to surrounding healthy tissues.^[^ ^17^ ^]^ A promising approach is the integration of MH with seed‐relied brachytherapy for tumor‐specific treatment. The main challenge in this approach lies in synchronizing the timing and balancing the dosage of MH with radiotherapy, as well as reducing the technical complexity involved in device integration, suggesting substantial space for development.^[^ ^18^ ^]^ Optimizing these aspects of combined therapies represents an urgent technical challenge that needs to be addressed in this field.^[^ ^19^ ^]^

In this study, we developed a localized, precise therapeutic strategy for brachytherapy combined with MH by synthesizing magnetic nanoparticles (MNPs), assembling them with ^125^I‐coated silver rods into composite seeds, and then simulating and validating the pancancer therapeutic potential of this approach. Additionally, through further biological characterization based on single‐cell transcriptomics, we explored the potential biological mechanisms underlying the anticancer effects of the composite seeds. Through this study, we present a promising new material and therapeutic method, providing novel insights for clinical cancer treatment.

## 2 Results

### 2.1 Construction and Physical Properties of ^125^I@MH Composite Seeds

**Figure 1:**
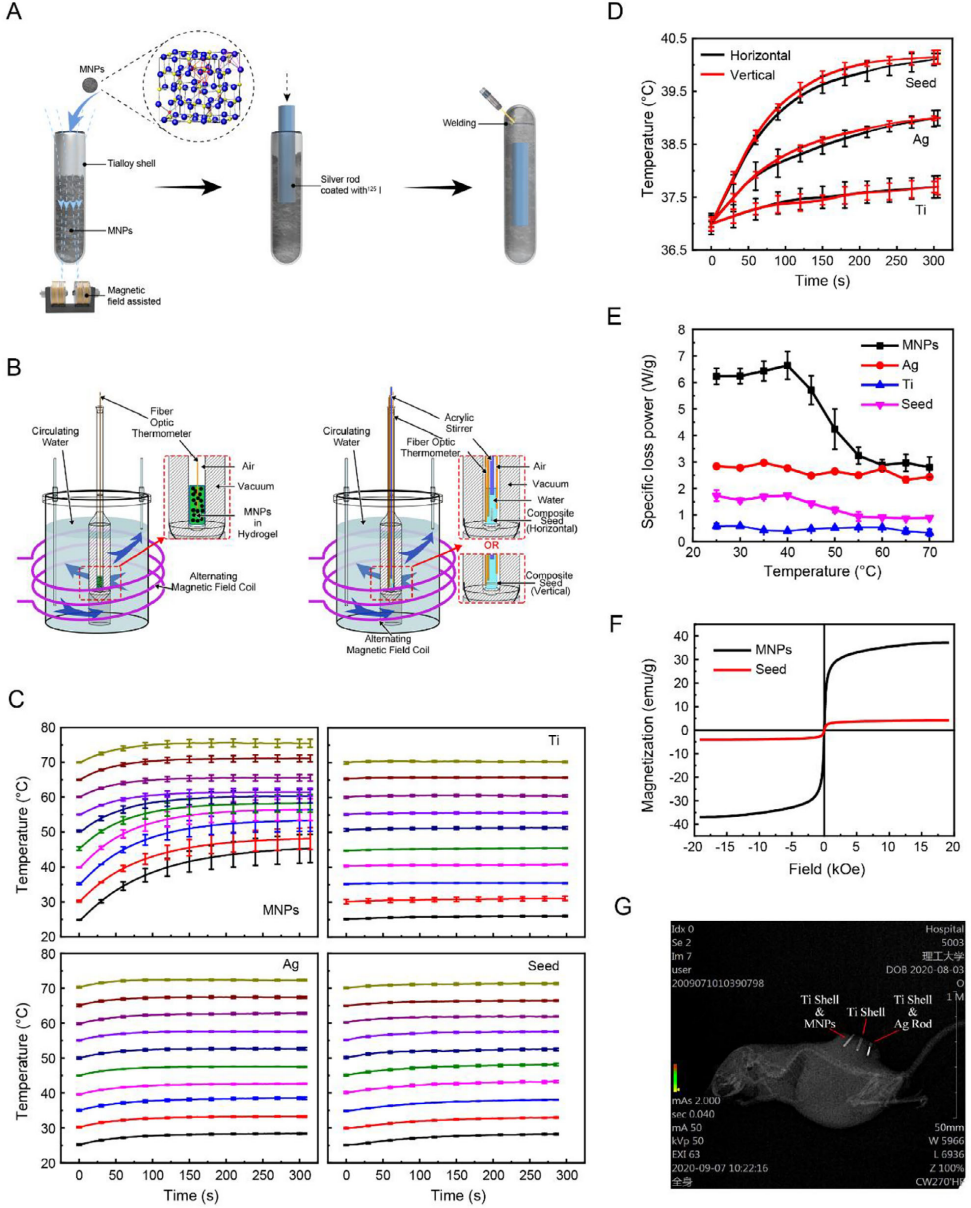
Construction and physical properties of ^125^I@MH composite seeds. A) Schematic diagram of composite seed assembly. B) Schematic diagram of the hydrogel‐based detection setup for heat generation efficiency. C) Heating curves of silver rods, titanium shells, magnetic nanoparticles (MNPs), and ^125^I@MH composite seeds at different temperatures. D) Heating curves representing the heat generation directionality of each component in the composite seed. E) Temperature‐dependent specific loss power (SLP) of each component in the composite seed. F) Magnetization curves of MNPs and composite seeds at room temperature. G) In vivo CT imaging of composite seeds. Data are presented as mean ± s.d.

Zn_0.54_Co_0.46_Cr_0.6_Fe_1.4_O_4_ MNPs were synthesized via a hydrothermal method using a protocol similar to that previously reported (for details, see Experimental Section – Self‐regulating magnetic nanoparticle synthesis).^[^ ^20^ ^]^ As shown in Figures S1–S4 (Supporting Information), the morphology, size distribution, and crystal structure of the synthesized Zn_0.54_Co_0.46_Cr_0.6_Fe_1.4_O_4_ MNPs were determined using transmission electron microscopy (TEM) and X‐ray diffraction (XRD). Figure S1 (Supporting Information) shows that the MNPs exhibit a tetragonal shape, and the size distribution can be fitted using Equation 1 (see Experimental Section), yielding an average size of 13.3 nm. Figure S2 (Supporting Information) presents the high‐resolution TEM results, indicating an interplanar spacing of 0.259 nm, corresponding to the (311) crystal plane of the cubic structure. Figure S3 (Supporting Information) shows the electron diffraction pattern, which reveals seven distinct diffraction rings. The XRD pattern in Figure S4 (Supporting Information) also reveals seven diffraction peaks corresponding to the crystal planes (220), (311), (400), (422), (511), (440) and (533) of the Fe_3_O_4_ standard Powder Diffraction Files (PDF) card (JCPDS‐ICDD database #88‐0866), which once again confirmed the cubic spinel structure of the MNPs. To determine the Curie temperature of the MNPs, the mass‒temperature curve was measured under an applied static magnetic field using thermogravimetric analysis (Figure S5, Supporting Information). The Curie temperature was identified as the temperature corresponding to the maximum value of the first derivative of this curve,^[^ ^21^ ^]^ which was determined to be 55°C (Figure S6, Supporting Information). The MNPs and a silver rod coated with ^125^I were assembled into a titanium shell to construct composite seeds (**Figure** **1**A; details see Experimental Section – Composite seed assembly; the physical image is shown in Figure S7, Supporting Information), which was then measured for its temperature‐dependent specific loss power (SLP) in a hydrogel‐based setup (Figure 1B; details see Experimental Section – Measurements of temperature‐dependent SLP).

The heating curves of the MNPs, silver rods, titanium shells, and composite seeds at different temperatures are shown in Figure 1C. The heat generation of the silver rod and titanium shell is minimally affected by the initial temperature. The heating curves in the orthogonal direction revealed that regardless of whether the sample axis was parallel or orthogonal to the magnetic field direction, the heating rate of the water remained similar (Figure 1D). After 10 min, the temperature on the surface of the composite seeds stabilized at 41.91±0.69°C and 41.40±0.74°C. As shown by the heat generation efficiency (Figure 1E), the heat generation efficiency of the silver rod and titanium shell remains almost unaffected by temperature, indicating a lack of self‐regulating thermal performance. In contrast, the heat generation efficiency of the composite seeds significantly decreases between 40–60°C, demonstrating temperature self‐regulating performance, which is attributed primarily to the self‐regulating properties of the MNPs.

The magnetization curve of the composite seed, measured using a vibrating sample magnetometer, is shown in Figure 1F, confirming that the magnetization originates primarily from the MNPs and is not affected by other components of the composite seeds.

Furthermore, the X‐ray scanning results revealed that the magnetic nanoparticles appeared relatively loose because of the gaps between the clusters of particles, resulting in an overall lower density than that of the silver rods (Figure 1G). The magnetic nanoparticles and the silver rod are distinguishable from the mouse skeleton in the grayscale image, demonstrating that the composite seed has X‐ray imaging tracking capability.

### 2.2 Simulation of Brachytherapy and Hyperthermia with ^125^I@MH Composite Seeds

**Figure 2:**
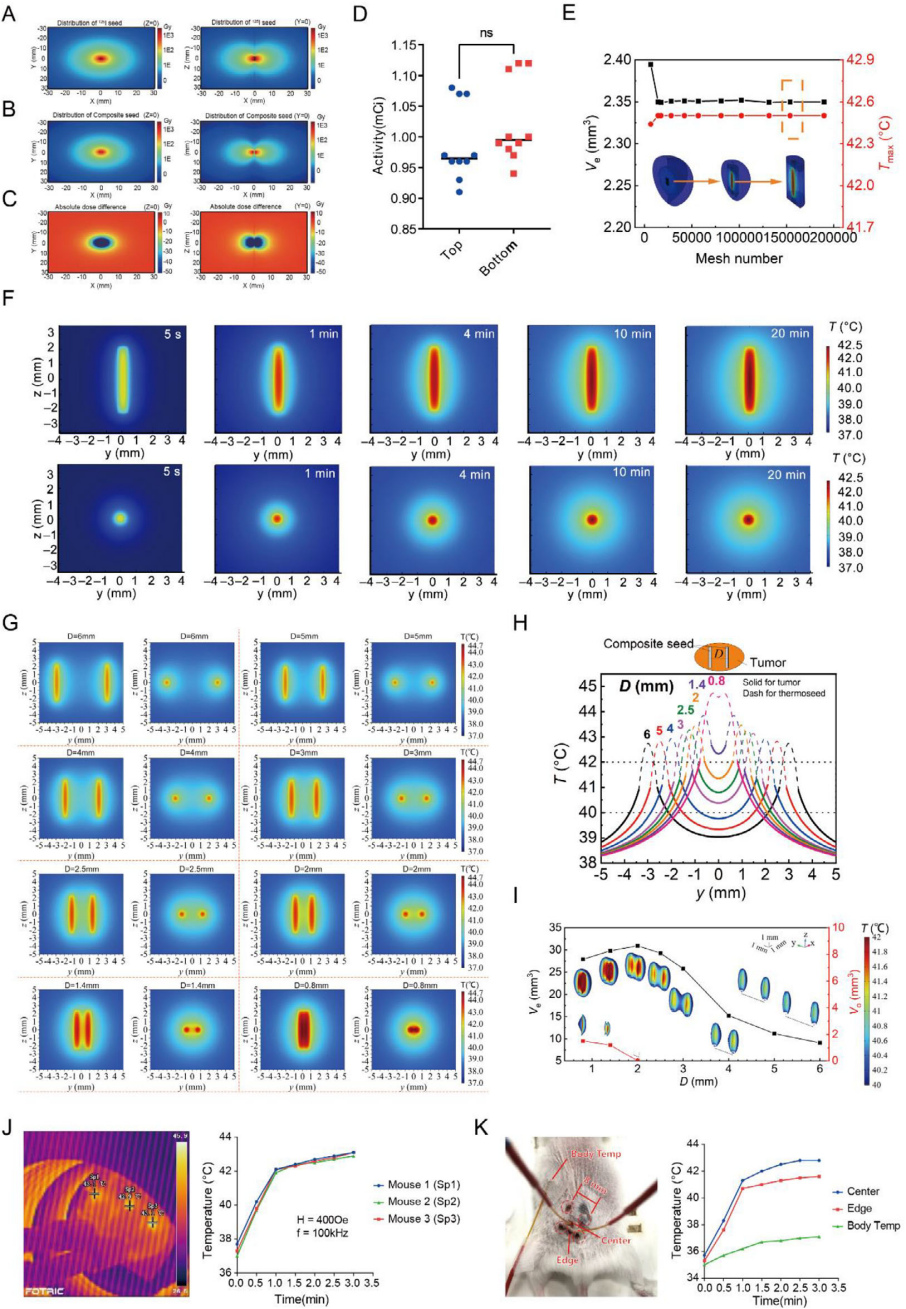
Simulation of brachytherapy and hyperthermia with ^125^I@MH composite seeds. A–C) Monte Carlo radiation dose simulation between ^125^I seeds and composite seeds. D) Radiation activity detection at the top and bottom of the composite seed. E) Convergence analysis of mesh numbers with respect to the effective therapeutic volume (Ve) and peak temperature (Tmax). F) Temperature contour figures of a single composite seed heating tumor in the transverse plane (upper) and the coronal plane (lower). G) Temperature contour figures of two composite seeds heating tumors at different distances. H) Temperature distributions in the tumor and composite seed along the central line of the coronal plane of the two composite seed cases. I) Effective therapeutic volume (V_e_) and overheating volume (V_o_) of tumors when two composite seeds are heated at different distances. The contour figures are half the volume of the corresponding tumor area. J) Heating performance of composite seeds in vivo. K) Heating conditions of composite seeds at different locations in vivo.

First, we performed radiation dose simulations. The dose distributions of composite and conventional seeds obtained through Monte Carlo simulation are shown in **Figure** **2**A,B, and the absolute dose differences between them are illustrated in Figure 2C. Compared with conventional methods, the inclusion of MNPs resulted in a significant reduction in the radiation dose, with the average dose at 1 cm from the seed center decreasing by 23.7%. Additionally, no significant difference was found in the radioactive activity between the bottom and top of the composite seed (Figure 2D).

Next, we checked the hyperthermia efficiency of the composite seed through COMSOL simulation. Since the temperature changes mainly occur around the seed for a single composite seed, the mesh number of a spherical area (radius: 4.5 mm) around it is considered for convergence analysis. As the mesh number in this area increases, the effective therapeutic volume (*V*_e_) of the tumor under hyperthermia (40–42 °C) and the peak temperature (*T*_max_) of the composite seed change, as shown in Figure 2E. A mesh number of 153,908 is selected. The heat is generated within the composite seed and dissipates into the surrounding tumor tissue, with the temperature increasing until it reaches equilibrium at ≈10 min. The peak temperature of 42.5 °C lies at the center of the composite seed. The maximum temperature of the tumor is 40.7 °C. The *V*_e_ is 2.35 mm^3^, slightly larger than a composite seed (Figure 2F).

In terms of two seeds, as the distance between the two parallel seeds decreased from 6 mm to 3 mm, the temperature fields around the two composite seeds overlapped each other more noticeably, increasing the temperature between them more observably (Figure 2G). The inner temperatures of the composite seeds also increased as the distance decreased (Figure 2G). The peak temperature always appears at the center of the composite seeds, which cannot directly exert effects on tumors (Figure 2H). The temperature distribution in the tumor requires further consideration. When the distance was 6 mm, a wide range of temperatures below 40 °C was observed between the composite seeds, which did not contribute to the effectiveness of the therapy (Figure 2H). When the distance is less than 2 mm, regions at temperatures greater than 42 °C appear, which threatens the health of the tissue when the tumor shrinks and leaves the composite seeds touching normal tissue (Figure 2H). The influences of distance are plotted in Figure 2I. When the distance is less than 2 mm, an overheated region appears near the edge of and between the two composite seeds. A further reduction in distance leads to a smaller effective therapeutic volume and a greater overheated volume. Thus, for the largest treatment area and to prevent overheating, the optimal distance between two composite seeds is ≈2 mm. Through parameter optimization, the maximum effective therapeutic volume is 30.83 mm^3^ at a distance of 2.05 mm when the overheating volume remains 0. We also simulate hyperthermia in four composite seeds (Figures S8–S10, Supporting Information). The maximum effective therapeutic volume was 166.30 mm^3^ and appeared at a distance of 5.85 mm without overheating. The proper arrangement of composite seed arrays can significantly enhance the therapeutic benefit of using only a single array.

We conducted hyperthermia simulations and in vivo experiments to further validate the heating capacity and stability of the composite seed. First, we simulated the hyperthermia process with COMSOL to imitate subcutaneous liver cancer tumors. Briefly, an ellipsoidal tumor with 1/5 minor axis embedded in healthy muscle tissue of body temperature was modeled (for details, see Experimental Section ‐ In vivo hyperthermia simulation). A natural convection condition of air was set on the surface of the tumor and tissue. The peak temperature of the composite seed is 44.2 °C at the center, and that of the edge is ≈42.7 °C (Figure S11, Supporting Information). The peak temperature on the tumor surface was 41.5 °C (Figure S11B, Supporting Information). Next, we implanted composite seeds into mice and performed heating experiments to verify the accuracy of the COMSOL simulation. Real‐time monitoring of the thermal response of the composite seeds in the subcutaneous tumors of the mice was conducted using both an infrared thermometer and an optical fiber thermometer (Figure 2J). Temperature measurements were taken at the center and edges of the composite seeds, as well as at the injection site (Figure 2K). The composite seeds heated rapidly in vivo, maintained a stable temperature, and exhibited excellent temperature control performance, with an effective heating range larger than that predicted by the COMSOL simulation.

### 2.3 ^125^I@MH Composite Seeds Enhance the Treatment Efficacy of Brachytherapy

**Figure 3:**
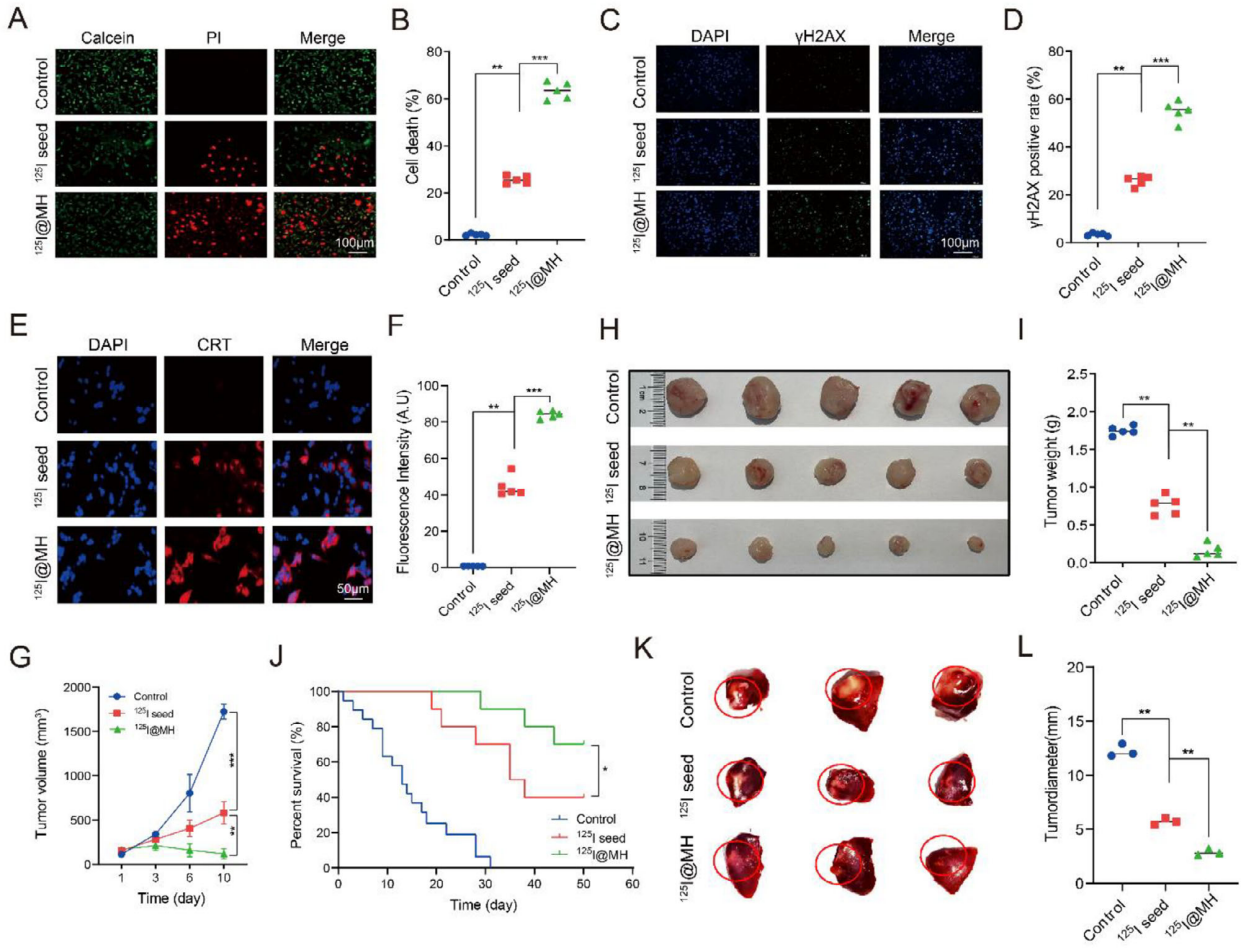
^125^I@MH composite seed enhances the treatment efficiency of brachytherapy. A,B) Representative immunofluorescence images of live/dead staining and comparisons of dead cell ratios in Hepa 1–6 cells under different treatment conditions: control group, ^125^I seed, and ^125^I@MH. Red fluorescence indicates dead cells (unpaired two‐sided *t*‐test, *n* = 5 per group). C,D) Representative immunofluorescence images of γ‐H2AX staining and comparisons of γ‐H2AX‐positive ratios in Hepa1–6 cells under different treatment conditions. Green fluorescence indicated γ‐H2AX positivity (unpaired two‐sided *t*‐test, *n* = 5 per group). E,F) Representative immunofluorescence images of CRT staining and comparisons of fluorescence intensity in Hepa 1–6 cells under different treatment conditions (unpaired two‐sided *t*‐test, *n* = 5 per group). G) Tumor growth curves of Hepa 1–6 subcutaneous tumors in mice after different treatments. Data are presented as mean ± s.d. (two‐way ANOVA, *n* = 5 per group). H,I) Pictures of harvested tumors and comparisons of tumor weights from Hepa 1–6 cell subcutaneous tumor‐bearing mice treated with different therapeutic methods (unpaired two‐sided t‐test, n = 5 per group). J) Survival curves of mice with Hepa 1–6 subcutaneous tumors after different treatments. K,L) Pictures of harvested tumors and comparisons of tumor diameters from N1S1 cell orthotopic tumor‐bearing rats after different treatments (unpaired two‐sided *t*‐test, 3 mice per group). ^*^: *P* < 0.05, ^**^: *P* < 0.01, ^***^: *P* < 0.001, ^****^: *P* < 0.0001.

We first tested the treatment efficacy of the composite seeds in vitro on liver cancer cells. The live/dead cell viability assays revealed significant differences in cell death rates across the treatment groups. Brachytherapy induced a notably greater percentage of cell death, while ^125^I@MH further enhanced cell death (**Figure** **3**A,B). The proportion of γ‐H2AX was elevated in the ^125^I group and further increased in the ^125^I@MH group (Figure 3C,D). The experimental results also revealed significant differences in the fluorescence intensity and localization of CRT (calreticulin) under different treatment conditions (Figure 3E,F). Specifically, the expression level of CRT in the control group was low, with fluorescence signals mainly localized within the cells. In contrast, in the ^125^I@MH group, CRT expression was significantly increased, with fluorescence signals predominantly distributed on the cell membrane. These differences indicate that ^125^I@MH treatment promotes immunogenic cell death (ICD). The significantly elevated level of CRT externalization in the ^125^I@MH group may be attributed to MH, which enhances cell membrane permeability and accelerates the transport of stress proteins, further promoting CRT externalization.

Next, we tested the anti‐liver cancer treatment efficacy of ^125^I@MH seeds through a Hepa 1‐6 mouse subcutaneous tumor model with three groups: the control, ^125^I, and ^125^I@MH groups. By comparing the growth velocities of the tumors and the weights of the harvested tumors on Day 10, we demonstrated that the tumor growth rates and final tumor weights in the ^125^I@MH group were significantly lower than those in the other two groups, with some tumors even exhibiting shrinkage (Figure 3I‒G; the details are shown in Figure S12, Supporting Information). The hematoxylin‐eosin (HE), Ki67, and terminal deoxynucleotidyl transferase‐mediated dutp nick‐end labeling (TUNEL) staining results demonstrated significant differences among the treatment groups, revealing fewer tumor cells (Figure S13, Supporting Information), decreased cell proliferation, and enhanced apoptosis in the ^125^I group. These effects were more pronounced in the ^125^I@MH group. Survival curves were generated (Figure 3J, *n* = 20) based on the criteria of either a tumor size exceeding 2000 mm^3^ or a survival time of 50 days. Notably, the survival time of the ^125^I@MH group was significantly longer than that of the control and ^125^I groups. The systemic safety of the composite seed was evaluated with the HE staining of the heart, liver, spleen, lungs, and kidneys, as well as the comparisons of weight growth curves, serum biochemical parameters (ALT, AST and creatinine) and complete blood counts (WBC, RBC and hemoglobin), which revealed no significant pathological abnormalities (Figure S14, Supporting Information). The same results were observed in the rat liver orthotopic tumor model, in which tumor growth was significantly suppressed in the ^125^I group and further inhibited upon the addition of MH (Figure 3K,L, Supporting Information). The results of Ki67 and TUNEL staining are shown in Figure S15 (Supporting Information) and were consistent.

We conducted similar in vivo experiments in mouse subcutaneous PCa models to verify whether the composite seeds exhibit similarly strong anticancer effects in other cancer types. As shown in Figure S16 (Supporting Information), the composite seeds demonstrated excellent pancancer antitumor effects.

### 2.4 Tumoral Heterogeneity and the TME Landscape in ^125^I and ^125^I@MH Therapy

**Figure 4:**
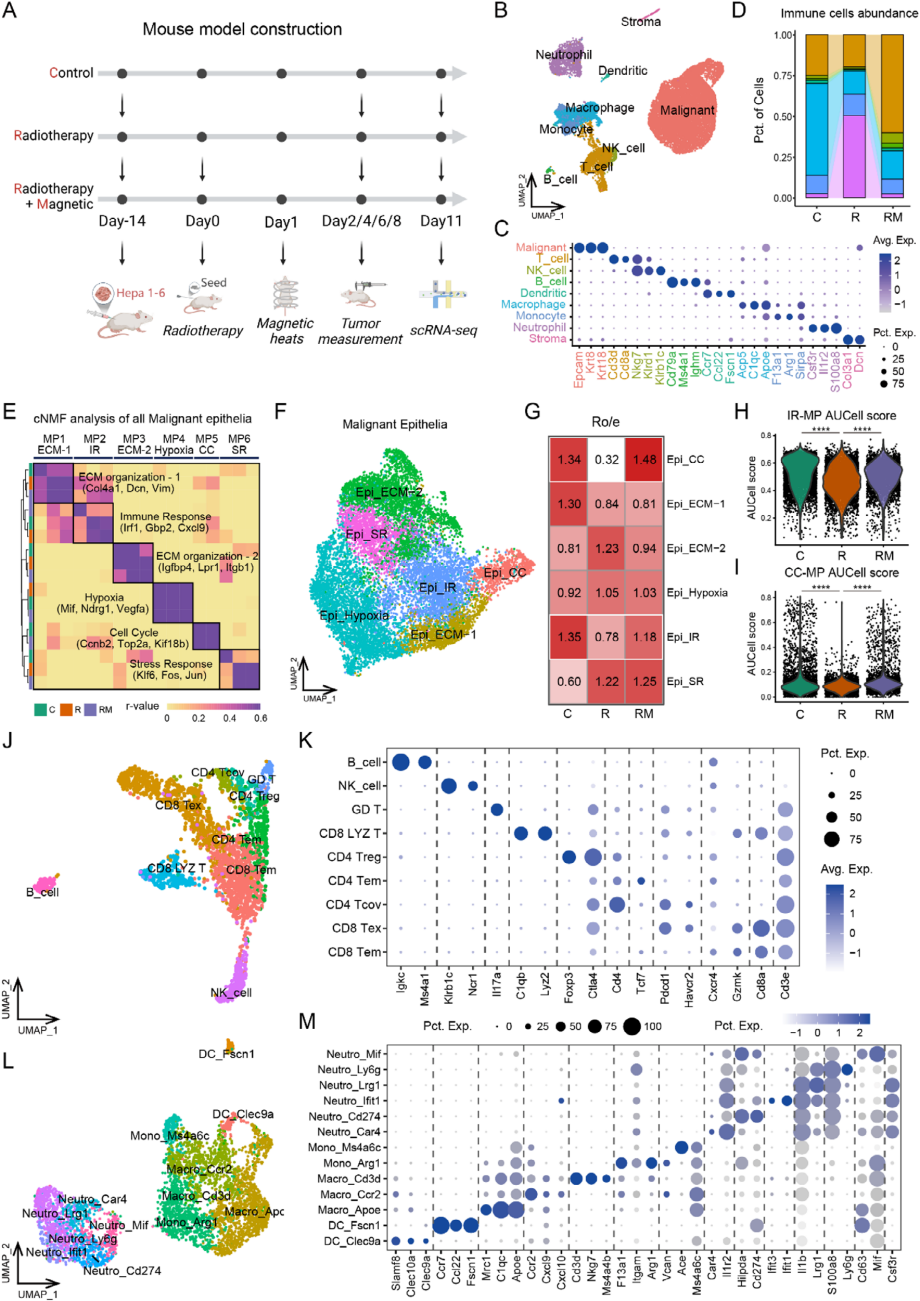
Tumoral heterogeneity and the TME landscape in ^125^I and ^125^I@MH therapy. A) Workflow for subcutaneous tumor implantation, ^125^I brachytherapy, MH, and scRNA‐seq in mice (1 mouse per group). B) UMAP plot of the single‐cell landscape, colored by general cell type. C) Dot plot showing the expression levels of well‐established markers for general cell types. D) Stacked bar chart showing the proportion of each cell type across different treatment groups. E) Heatmap showing the pairwise correlations of 17 intratumoral programs derived from malignant epithelial cells among samples from different treatment groups, from which 6 coherent meta‐programs (MPs) were identified. F) UMAP plot of malignant epithelial cells, colored by MP‐marked cell types. G) Ro/e plot showing the distribution of different types of malignant epithelial cells across different treatment groups. H,I) Violin plots of AUCell scores for the IR‐MP (H) and CC‐MP (I) of malignant epithelial cells, comparing intergroup differences (Kruskal‐Wallis test). J) UMAP plot of lymphocytes, colored by cell subtype. K) Dot plot showing the expression levels of markers for different lymphocyte subtypes. L) UMAP plot of myeloid cells, colored by cell subtype. M) Dot plot showing the expression levels of markers for different myeloid cell subtypes. ^*^: *P* < 0.05, ^**^: *P* < 0.01, ^***^: *P* < 0.001, ^****^: *P* < 0.0001.

To investigate the underlying mechanism of enhanced tumor elimination using ^125^I@MH therapy, we constructed mouse models subjected to different treatment strategies [the control (C), ^125^I radiotherapy/brachytherapy (R), and ^125^I@MH therapy (RM) groups] and harvested tumor tissues for scRNA‐seq (**Figure** **4**A). Nine general cell types were identified with established markers (Figure 4B,C). Intriguingly, neutrophils were significantly enriched in the R group but downregulated when MH was added. In contrast, the abundance of T cells and macrophages, especially T cells, was reduced in the R group while recused in the RM group (Figure 4D). This phenomenon suggested that immune reconstruction might significantly improve the treatment outcomes of ^125^I@MH therapy compared with ^125^I brachytherapy alone.

We subsequently investigated how malignant cells remodel their lineage and interact with such immune TME alterations. Consensus nonnegative matrix factorization (cNMF) analysis extracted six meta‐programs (MPs) from the three samples, which were assigned as extracellular matrix organization–1 (ECM‐1‐MP; *Col4a1* and *Dcn*), immune response (IR‐MP; *Irf1* and *Gbp2*), ECM organization–2 (ECM‐2‐MP; *Igfbp4* and *Lpr1*), hypoxia (hypoxia‐MP; *Mif* and *Ndrg1*), the cell cycle (CC‐MP; *Ccnb2* and *Top2a*), and the stress response (SR‐MP; *Klf6* and *Fos*), which correspond to different malignant cell subclusters (Figure 4E,F). Consistent with previously reported biological processes in brachytherapy resistance,^[^ ^3^ ^]^ ECM‐2 and hypoxia epithelial clusters were enriched, whereas CC and IR clusters were diminished in the R group. Notably, these changes were reversed by MH (Figure 4G). We also detected a pronounced difference in the expression of IR‐MPs between the treatment groups; IR‐MPs were significantly downregulated in the R group but rescued in the RM group (Figure 4H). This trend was not dramatic for CC‐MP (Figure 4I). Taken together, malignant epithelial cells experienced immune evasion following ^125^I brachytherapy, and this immune evasion can be rescued by ^125^I@MH treatment, which is consistent with the immune TME reconstruction described in the previous section. Furthermore, we annotated lymphocytes and myeloid cells at a higher resolution, identifying seven T‐cell subtypes, one NK cell type, one B‐cell type, two dendritic cell subtypes, five macrophage subtypes, and six neutrophil subtypes, for further investigation of the reprogramming of the immune TME (Figure 4J–M).

### 2.5 ^125^I brachytherapy‐Induced *Cd274* ^+^ Neutrophil Recruitment Promotes Immune Escape

**Figure 5:**
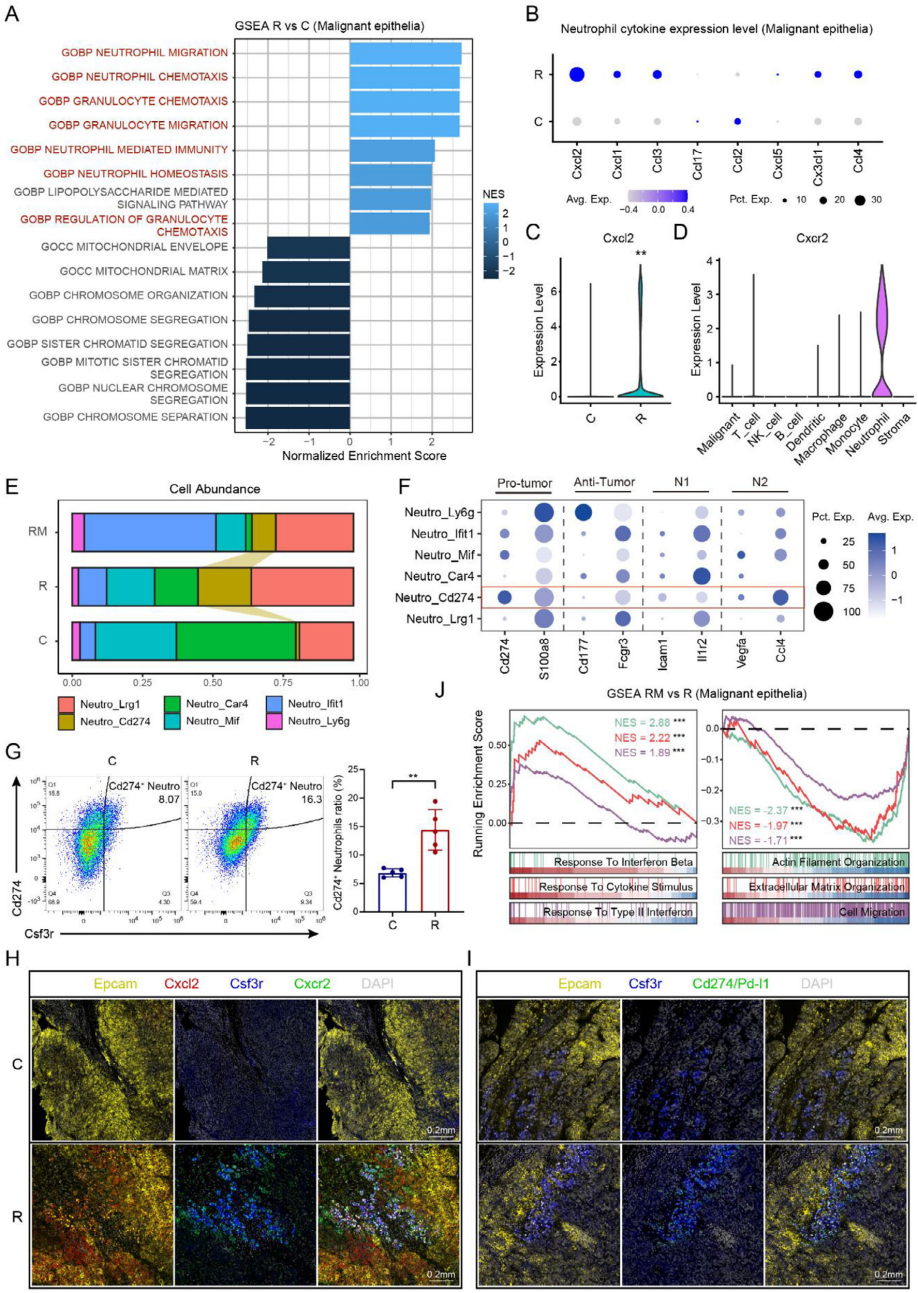
^125^I brachytherapy‐induced *Cd274* ^+^ neutrophil recruitment promotes immune escape. A) Bar plot of the GSEA results showing functional differences in malignant epithelial cells between the radiotherapy/brachytherapy (R) and control (C) groups, with the bar length representing the normalized enrichment score. B) Dot plot showing differential expression levels and proportions of neutrophil chemokine‐related genes between the R and C groups. C) Violin plot of the differential expression of *Cxcl2* between the R and C groups (Kruskal‐Wallis test). D) Violin plot showing the differential expression of *Cxcr2* across general cell types. E) Stacked bar chart showing the proportions of neutrophil subtypes across different treatment groups. F) Dot plot illustrating the expression levels and proportions of functional markers in neutrophil subtypes. G) Flow cytometry analysis of subcutaneous tumors comparing the proportion of Cd274+ neutrophils between the C and R groups. Data are presented as mean ± s.d. (unpaired two‐sided t‐test, *n* = 5 per group). H) Representative images of multiplex immunofluorescence (mIF) staining showing colocalization of Cxcl2‐Cxcr2 interactions between malignant epithelial cells and neutrophils in subcutaneous tumors in the R and C groups (5 mice per group). I) Representative images of mIF staining comparing differential infiltration levels of *CD274* ^+^ neutrophils in subcutaneous tumors between the R and C groups (5 mice per group). J) GSEA showing functional differences in malignant epithelial cells between the ^125^I@MH (RM) and R groups. ^*^: *P* < 0.05, ^**^: *P* < 0.01, ^***^: *P* < 0.001, ^****^: *P* < 0.0001.

Since the IR‐MP was downregulated in the R group, we compared the function of malignant epithelial cells through gene set enrichment analysis (GSEA) (**Figure** **5**A). As expected, multiple pathways related to neutrophil recruitment were activated in malignant cells in the R group. Therefore, we tested the expression levels of neutrophil chemokines and found that *Cxcl2* was highly and extensively expressed in the malignant cells of R but not in those of the C group (Figure 5B,C). The receptor of *Cxcl2*, *Cxcr2*, was identified specifically in neutrophils, suggesting that malignant cells recruit neutrophils through Cxcl2–Cxcr2 interactions (Figure 5D).

We subsequently investigated the evolution of neutrophils during treatment. Among the neutrophil subtypes, the number of *Cd274^+^* neutrophils tended to increase in the R group but decreased in the RM group, with a notable change (Figure 5E). Cd274, the gene encoding Pd‐l1, is a well‐established immune‐suppressive gene related to T‐cell exhaustion and has been clinically used as a target for immune checkpoint blockade. Further annotation of neutrophil subtypes with functional markers revealed that *Cd274* ^+^ neutrophils highly expressed protumor (*Cd274* and *S100a8*) and N2 (*Vegfa* and *Ccl4*) markers, suggesting that *Cd274* ^+^ neutrophils are typical polymorphonuclear myeloid‐derived suppressor cells (PMN‐MDSCs) (Figure 5F).^[^ ^22^, ^23^ ^]^ MDSCs promote immune evasion during brachytherapy, partially through hindering the antitumor functions of T cells.^[^ ^4^, ^24^ ^]^ We additionally applied flow cytometry to validate the increased proportion of *Cd274* ^+^ neutrophils in the R group (Figure 5G; for detailed results, see Figure S17, Supporting Information). mIF staining revealed colocalization of Cxcl2‐Cxcr2 interactions between malignant cells and neutrophils and enrichment of *Cd274* ^+^ neutrophils in the R group (Figure 5H,I).

Next, we compared the functional differences in malignant epithelial cells between the R and RM groups to confirm whether MH can indeed reverse the immune evasion induced by ^125^I brachytherapy. As expected, immune response signaling pathways, including those related to the response to interferon and cytokine stimuli, were activated in the RM group. In contrast, ECM organization‐ and cell migration‐related pathways were upregulated in the R group (Figure 5J,K). Therefore, we further investigated how MH reversed the suppressive immune TME caused by ^125^I brachytherapy.

### 2.6 MH Rescues Immune Escape Induced by ^125^I Brachytherapy

**Figure 6:**
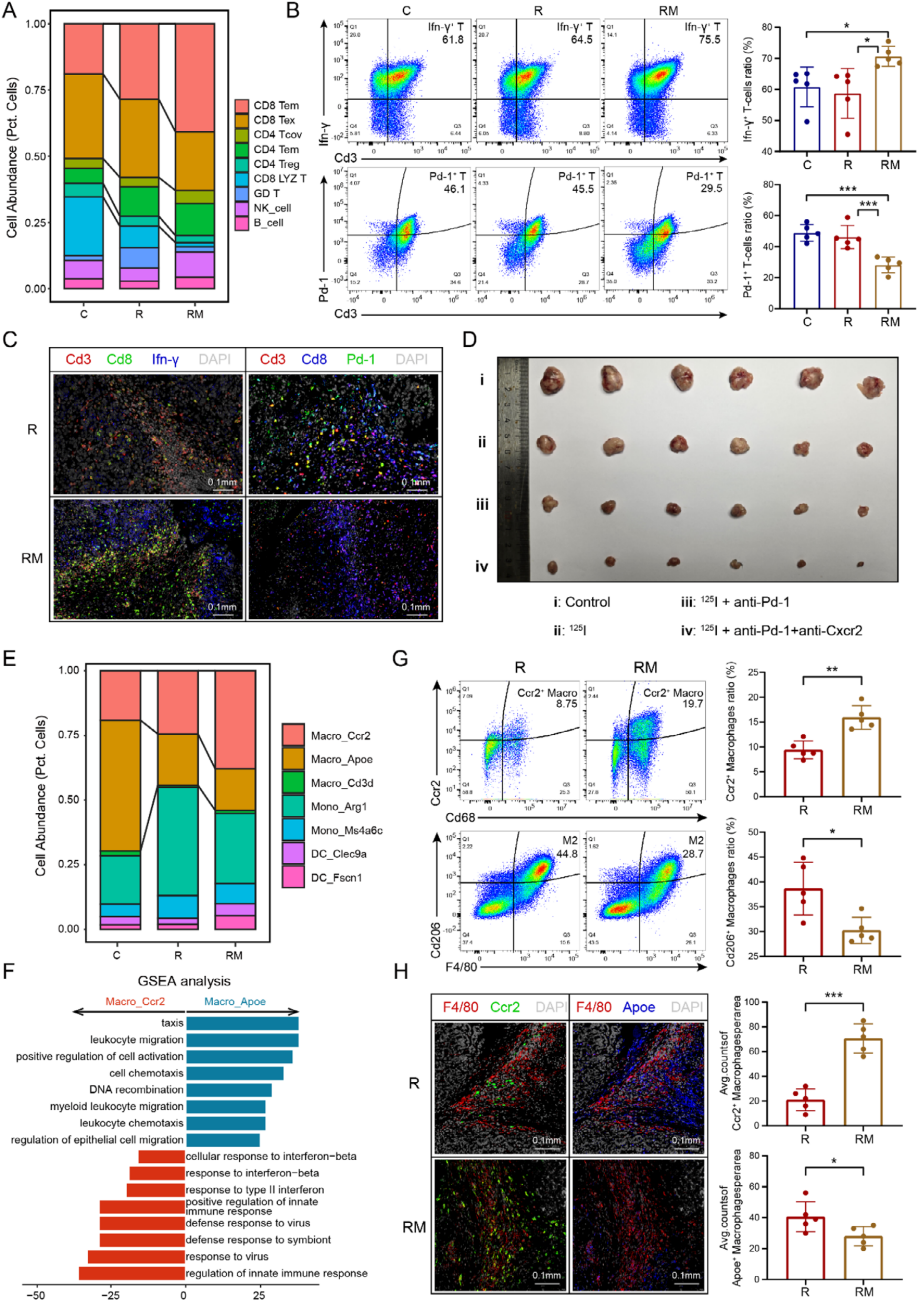
MH rescues immune escape induced by ^125^I brachytherapy. A) Stacked bar chart showing the proportions of lymphocyte subtypes across different treatment groups. B) Flow cytometry analysis of subcutaneous tumors comparing the proportions of exhausted T cells (upper) and effector T cells (lower) among the C, R, and RM groups. Data are presented as mean ± s.d. (unpaired two‐sided *t*‐test, *n* = 5 per group). C) Representative image of multiplex immunofluorescence (mIF) staining comparing differential infiltration levels of exhausted T cells (upper) and effector T cells (lower) in subcutaneous tumors between the RM and R groups (5 mice per group). D) Comparison of subcutaneous tumor sizes in mice following no treatment, ^125^I treatment, ^125^I + anti‐PD‐1 treatment, and ^125^I + anti‐PD‐1 + anti‐Cxcr2 treatment (6 mice per group). E) Stacked bar chart showing the proportions of phagocyte subtypes across different treatment groups. F) Bar plot of the GSEA results showing functional differences between *Apoe* ^+^ macrophages and *Ccr2* ^+^ macrophages, with the bar length representing the normalized enrichment score. G) Flow cytometry analysis of subcutaneous tumors comparing the proportions of *Ccr2* ^+^ macrophages (upper) and *Apoe* ^+^ macrophages (lower) among the C, R, and RM groups. Data are presented as mean ± s.d. (unpaired two‐sided *t*‐test, *n* = 5 per group). H) Representative image of mIF staining comparing the differential infiltration levels of *Ccr2* ^+^ macrophages (upper) and *Apoe* ^+^ macrophages (lower) in subcutaneous tumors between the RM and R groups. Data are presented as mean ± s.d. (unpaired two‐sided *t*‐test, *n* = 5 per group). ^*^: *P* < 0.05, ^**^: *P* < 0.01, ^***^: *P* < 0.001, ^****^: *P* < 0.0001.

Considering that T cells are the main target cells of Pd‐l1, we checked the cell abundance of different lymphocytes between the groups. An increase in effector memory T (T_em_) cells and a decrease in exhausted T (T_ex_) cells were found in the RM group compared with those in the R group, which is consistent with the aforementioned conclusion that MH might rescue the T‐cell exhaustion status induced by Pd‐l1 and Pd‐1 interactions (**Figure** **6**A). Similarly, we validated the distribution features of T_em_ and T_ex_ cells in the RM and R groups by labeling Pd‐1 and Ifn‐γ via flow cytometry and mIF staining (Figure B‐C; for detailed results, see Figures S18 and S19, Supporting Information).

These findings prompted us to explore whether immunotherapy could also enhance the efficacy of ^125^I brachytherapy. Therefore, we simulated anti‐Pd‐1 and anti‐Cxcr2 therapy in vivo and found that anti‐Pd‐1 therapy could improve the outcome of ^125^I brachytherapy and that anti‐Cxcr2 therapy could further enhance the efficacy (Figure 5D). This compelling result indicated that Cd274^+^ neutrophils and T_ex_ cells mediated the immune evasion induced by ^125^I brachytherapy to some extent and that MH could be used as a substitute for immunotherapy to rescue this effect.

Moreover, we explored lineage changes in phagocytes. Like *Cd274* ^+^ neutrophils, a monocytic MDSC (M‐MDSC) subtype, *Arg1* ^+^ monocytes, which overexpress *Arg1* and *Cd11b (Itgam)*, were upregulated only in the R group (Figures 1M and 6E). In terms of macrophages, *Apoe* ^+^ macrophages and *Ccr2* ^+^ macrophages exhibited opposite changes in the RM group compared with those in the R group (Figure 6E). *Apoe* ^+^ macrophages, which express *Cd206* (*Mrc1)* and *C1qc*, have been defined as typical tumor‐associated macrophages (TAMs) with protumor functions in previous studies.^[^ ^25^, ^26^ ^]^ In contrast, *Cxcl9* and *Cxcl10*, which are well‐known markers of M1 and proinflammatory differentiation,^[^ ^27^, ^28^ ^]^ were found to be upregulated in *Ccr2* ^+^ macrophages (Figure 1M). The GSEA of Apoe^+^ and Ccr2^+^ macrophages validated the functional differences between these subtypes (Figure 6F). Macrophages are important antigen‐presenting cells (APCs) that play crucial roles in activating T‐cell‐mediated immunity. The shift in the ratio of *Ccr2* ^+^ macrophages to *Apoe* ^+^ macrophages, which was also validated through flow cytometry and mIF staining (Figure G‐H; for detailed results, see Figures S20–S21, Supporting Information), may represent an alternative mechanism by which RM therapy rescues T‐cell immune evasion mediated by ^125^I brachytherapy.

In summary, under ^125^I brachytherapy, *Cd274* ^+^ neutrophils, as the predominant suppressive immune cells, mediate T‐cell immune evasion, leading to resistance to brachytherapy. MH reactivated T‐cell immunity, rescuing resistance to treatment and enhancing brachytherapy outcomes.

### 2.7 Pancancer Immune Reconstruction Triggered by ^125^I@MH Therapy

We also preliminarily explored the immune remodeling effects of ^125^I@MH therapy by simulating composite seed‐mediated brachytherapy and MH in PCa. Through scRNA‐seq analyses of subcutaneous tramp‐c2 tumors derived from mice that underwent sham therapy, ^125^I brachytherapy or ^125^I@MH therapy, we observed an increased proportion of T cells following treatment (Figure S22A,B, Supporting Information). For T cells, similar to findings in liver cancer, the immune activity of T cells was enhanced after treatment, particularly following ^125^I@MH therapy. This was evidenced by a decrease in the proportion of Tex cells and an increase in the proportion of Cd8^+^ T_em_ cells (Figure S22C,D, Supporting Information). For phagocytes, the changes in the proportions of M1‐differentiated *Cxcl10* ^+^ and M2‐differentiated *Apoe* ^+^ macrophages further supported the activation of tumor immunity by ^125^I@MH therapy. A proinflammatory *Nlrp3* ^+^ tissue‐resident macrophage (TRM) population was also activated following treatment (Figure S22E,F, Supporting Information). We further validated the decrease in the proportion of T_ex_ cells after ^125^I@MH therapy by flow cytometry (Figure S22G,H; for detailed results, see Figure S23, Supporting Information).

Collectively, through analyses of the treatment‐induced TME in PCa, we found that, despite employing distinct approaches, composite seed‐based ^125^I@MH therapy enhanced the antitumor immunity of T cells, primarily by increasing the proportion of effector T cells, thus improving the treatment efficacy of seed‐based brachytherapy.

## 3 Discussion

Herein, the Zn_0.54_Co_0.46_Cr_0.6_Fe_1.4_O_4_ MNPs with low Curie temperature were assembled with ^125^I‐coated silver rods into a titanium shell to form a novel composite seed with the functions of both brachytherapy and MH. This composite seed has the following characteristics: i) The energy production efficiency is independent of the direction angle of the millimeter‐hybrid with respect to the magnetic field. ii) The hyperthermia temperature can be self‐regulated. iii) The shape and size enable mass production within the current industry procedure.

To integrate hyperthermia and brachytherapy, the MNPs/radioactive elements nano‐hybrids, such as ^103^Pd/Fe‐oxide, ^161^Tb/gold/γ‐Fe_2_O_3_, and ^111^In/dextran /iron oxide, have been proposed for intratumoral administration. Yet, the migration, loss, and redistribution of nano‐hybrids in the tumor are inevitable due to the release of intracellular solution in therapy, resulting in the uneven temperature field in the tumor after multiple hyperthermia.^[^ ^29^ ^]^ The composite seed invented here is capable of providing brachytherapy and hyperthermia simultaneously to guarantee the synergistic treatment effect.^[^ ^30^ ^]^ Through the design of composite seed structures, the issues of magnetic hyperthermia nanoparticle loss and poor accumulation have been resolved. This approach integrates two therapies into a single treatment platform, eliminating the need for multiple surgeries. The major heat‐producing component, MNPs, relies on relaxation loss and hysteresis loss to generate heat rather than the eddy current loss of conventional ferromagnetic thermoseeds.^[^ ^31^ ^]^ Thus, the heat production ability of the composite seed is not affected by the direction of the magnetic field, leading to a more stable and controllable temperature increase in the tumor. Compared with traditional heating methods, which cause MNPs to dissipate within tumors, composite seeds concentrate heat in the target area with high efficiency and strong targeting ability, protecting surrounding tissues.

Furthermore, our composite seeds, which benefit from self‐regulating MNPs, reduce the heating efficiency by 32% when the temperature increases from 40 to 50 °C, significantly lowering the risk of overheating and providing stable and controllable MH. The impact of composite seeds on radiation dosage can be addressed early by increasing the radionuclide input according to Monte Carlo simulation results. In the future, we can further integrate microelectronic and sensing technologies to achieve intelligent parameter settings for both hyperthermia and brachytherapy, enabling precise and adaptive treatment control. Additionally, nanotechnology can be utilized to modify shells with advanced features such as heat‐driven drug release, transforming composite seeds into a multifunctional therapeutic platform capable of supporting multimodal treatments. The structure and treatment parameters of brachytherapy rods can be tailored based on the specific tumor type and the patient's physiological characteristics, paving the way for personalized and optimized cancer treatments.

Using in vivo experiments, we discovered that the underlying mechanism of the improved therapeutic effect might involve the rescue of T‐cell immune exhaustion induced by radiation therapy by MH. Specifically, brachytherapy induced the recruitment of Cd274 (Pd‐l1)‐positive neutrophils, which are a type of protumor PMN‐MDSC. Cd274^+^ neutrophils play a critical role in malignancy progression by inducing immune suppression in various cancers,^[^ ^32^, ^33^, ^34^ ^]^ including liver cancer.^[^ ^35^ ^]^ This neutrophil subtype has also been associated with resistance to lenvatinib and PD‐1 inhibitor therapies in liver cancer.^[^ ^36^, ^37^ ^]^ In terms of brachytherapy, off‐target radiation exposure to normal tissues and cells during treatment led to the accumulation of neutrophils in breast cancer; blocking degranulation significantly offset this effect.^[^ ^38^ ^]^ These studies support our findings that brachytherapy in liver cancer promotes immune escape and leads to treatment resistance by recruiting neutrophils. Additionally, we found that the recruitment of neutrophils relies on Cxcl2–Cxcr2 interactions. A recent study revealed that tumor‐initiating cells specifically recruit tumor‐promoting neutrophils via the CXCL2‐CXCR2 axis and create an immunosuppressive TME in hepatocellular carcinoma, supporting our findings.^[^ ^39^ ^]^

Combined with MH through the composite seed, the immune escape conditions induced by brachytherapy could be reversed through a decrease in the proportion of Pd1+ exhausted T cells and an increase in Ifn‐γ+ effector T cells. Hyperthermia was proven to increase CD8^+^/IFN‐γ^+^ T cells and CD8^+^/TNF‐α^+^ T cells and enhance the immune function of CD8^+^ T cells, thus converting “cold” tumors into “hot” tumors.^[^ ^40^ ^]^ The findings of this study provide a new perspective on the biological mechanisms underlying the sensitization of brachytherapy by hyperthermia, further supporting the necessity of developing combination therapies. Furthermore, apart from liver cancer, we found that the composite seeds promoted the response to brachytherapy in PCa through distinct approaches but similar T‐cell activation effects. Overall, by strengthening T‐cell‐derived adaptive immunity, ^125^I@MH‐based combination therapy with composite seeds has the potential for clinical treatment of multiple cancers.

Prospectively, our preclinical study explores a combined brachytherapy‐hyperthermia approach for tumors, which might work in clinic following the protocol involves: 1) **Pre‐plan**—using CT/MRI/sonography data of tumor to simulate seed placement, radiation dose, and magnetic hyperthermia parameters; 2) **Implantation**—image‐guided percutaneous seed placement, allowing the brachytherapy last for 3–4 half‐lives of ^125^I; 3) **Post‐implantation analysis**—rescanning to simulate and adjust hyperthermia settings; and 4) **Hyperthermia**—applying a magnetic field (e.g., 400 Oe, 100 kHz) while monitoring vital signs and patient feedback. The composite seeds are designed as permanent implants with excellent tissue compatibility. Patients would undergo treatment in a clinical environment (e.g., radiation oncology or interventional suite), likely a few times per week, depending on tumor location and clinical goals.

In conclusion, controllable‐temperature magnetic hyperthermia‐radiation composite seed represents a cutting‐edge advancement in nanoscience and multimodal cancer therapy, resulting in a high degree of innovation. By enabling precise temperature control, multifunctional integration in the future, and optimization of biosafety, composite seeds are expected to play a critical role in cancer treatment and lay the foundation for developing multimodal combination therapy platforms. Future research should focus on clinical translation and safety evaluation to facilitate the transition of this technology into practical applications.

## 4 Experimental Section

### Self‐Regulating Magnetic Nanoparticle Synthesis

The Zn_0.54_Co_0.46_Cr_0.6_Fe_1.4_O_4_ MNPs were synthesized via the hydrothermal method by using a similar protocol in the literature.^[^ ^20^ ^]^ A clear solution was prepared by dissolving 8.91 mmol of ZnCl_2_, 7.59 mmol of CoCl_2_·6H_2_O, 9.9 mmol of CrCl_3_·6H_2_O, and 23.1 mmol of FeCl_3_·6H_2_O in 80 mL of deionized water under stirring. Subsequently, 150 mL of 4 mol/L NaOH solution was added dropwise into the metal salt solution while stirring magnetically at room temperature. The resulting mixture was then transferred to a sealed autoclave and subjected to hydrothermal treatment at 250°C for 6 h with a stirring speed of 200 rad·min^−1^. After completion, the autoclave was allowed to cool naturally to room temperature. The resulting black powder was washed with deionized water and ethanol until a neutral pH was achieved and then dried at 80°C for 12 h. The raw materials, including zinc chloride (ZnCl_2_, ≥98%), cobalt chloride hexahydrate (CoCl_2_·6H_2_O, ≥99%), chromium chloride hexahydrate (CrCl_3_·6H_2_O, ≥99%), ferric chloride hexahydrate (FeCl_3_·6H_2_O, ≥99%), and sodium hydroxide (NaOH, ≥96%), were sourced from Shanghai Aladdin Biochemical Technology Co., Ltd., China. Ethanol (≥99.7%) was provided by Tianjin Fuyu Fine Chemical Co., Ltd., China. All reagents were used directly without further purification.

### Composite Seed Assembly

A magnet was placed at the bottom of a titanium shell (3.6 ± 0.8 mg, 5.3 ± 0.04 mm in length, outer diameter 0.8 ± 0.02 mm) to attract 0.6 mg of MNPs into the shell via magnetic force. The titanium shell containing MNPs and a silver rod coated with ^125^I (6.0 ± 0.6 mg, 3.0 ± 0.04 mm in length, outer diameter 0.5 ± 0.02 mm) were clamped together using negative pressure for automated assembly. Subsequently, an additional 0.6 mg of MNPs was loaded into the shell through magnetic guidance. Finally, the opening of the titanium shell was sealed using a pulsed micro‐beam plasma arc welding machine (LHM8‐50, Jiangsu Dade Heavy Industry Co., Ltd., China), resulting in a composite seed (Figure 1A; Figure S24, Supporting Information,10.8 ± 1.02 mg, 4.5 ± 0.3 mm in length, outer diameter 0.8 ± 0.02 mm). All assembly processes were conducted in a radiation‐shielded lead glove box. The titanium shell, silver rod, and assembly site were provided by Junan Pharmaceutical Technology Co., Ltd., Ningbo, China.

### Measurements of Temperature‐Dependent SLP

First, the heating curves of magnetic nanoparticles and other components in the composite seed at different initial temperatures were measured. Then, the heat generation efficiency was calculated from the heating curves to obtain the relationship between heat generation efficiency and temperature. The experimental setup is shown in Figure 1B. The test tubes in the experimental setup were different for MNPs and composite components. For MNPs, they were uniformly dispersed in the hydrogel to avoid the influence of spatial concentration differences on the measured heat generation efficiency.^[^ ^41^ ^]^ Two milliliters of magnetic nanoparticle hydrogel dispersion were placed in a centrifuge tube, which was then placed inside a Dewar vacuum flask to reduce heat dissipation and minimize its impact on the calculation of particle heat generation efficiency. One fiber optic thermometer was inserted into the test tube to monitor the temperature. For composite seed components, the silver rod, the titanium shell, and the overall composite seed were included. In each experiment, eight same samples were fixed either parallel or perpendicular to the magnetic field direction along their axial direction in the test tubes. Three fiber optic thermometers were inserted from near to far around the center of the sample array. During the test, 2 mL of deionized water in the test tube was stirred using a mechanical stirrer with an acrylic stirring rod to accelerate convective heat dissipation, ensuring that the temperature differences of the three thermometers remained within ±0.2°C, thereby reducing errors in the calculation of heat generation efficiency caused by temperature gradients. Both setups were then immersed in a temperature‐controlled circulating water system. When the sample temperature stabilized at the target initial temperature, the alternating magnetic field generator (GUF‐30T, Shenqiu Yongda High‐Frequency Equipment Co., Ltd., Henan, China) was turned on. The samples were heated under conditions of 400 ± 5 Oe and 100 ± 5 kHz for 3 min, and the temperature‐time curves of the samples were recorded. The initial temperature was set from 25 to 75°C, with an interval of 5°C. At each initial temperature, measurements were repeated four times, and the heating curves were used to calculate the heat generation efficiency.

Based on the obtained relationship between temperature and time, the SLP could be calculated to describe the amount of heat converted per unit mass of magnetic medium per unit time. The calculation formula was shown in Equation (1).^[^ ^42^ ^]^                  

(1) $$SLP=c_{\mathrm{pMHG}}\left(\frac{dT}{dt}\right)\left(\frac{m_{\mathrm{MHG}}}{m_{\mathrm{MNP}}}\right)\;$$

Where *c*_pMHG_ was the specific heat capacity of the magnetic nanoparticle dispersion (2.30 ± 0.18 J/(g·°C)), measured using differential scanning calorimetry as referenced in the literature^[^ ^43^, ^44^ ^]^; *T* was the temperature (°C); t was the time (s); $\frac{dT}{dt}$ was the initial slope of the temperature‐time curve during the initial heating period, when heat loss can be neglected; *m*_MHG_ was the total mass of the dispersion; and *m*_MNP_ was the mass of the magnetic nanoparticles.

### Imaging Tracking Performance

To evaluate the imaging tracking performance of the composite seed, titanium shells containing magnetic nanoparticles, empty titanium shells, and titanium shells containing silver rods commonly used in brachytherapy were implanted into the tumors on the backs of mice. X‐ray scans were performed using a CT scanner (Apsaras CT, Konda Intercontinental Medical Devices Co., Ltd., Ningbo, China).

### Radiation Dose Simulation

Monte Carlo simulation software GATE was used to simulate the radiation dose distributions of composite seeds and conventional seeds (^125^I seeds), for evaluating the impact of magnetic nanoparticles. The CAPRAC‐T well counter was utilized to measure the radioactive activity at both the bottom and top of composite seeds, in order to evaluate potential differences in activity between these two locations.

### Deep‐Seated Tumor Hyperthermia Simulations

The models were shown in Figure S25 (Supporting Information), *i*.*e*., the composite seeds (diameter: 0.80 ± 0.02 mm, length: 4.50 ± 0.30 mm) were embedded at the center region of a sphere tumor (15 mm radius), surrounded by healthy liver tissue (25 mm radius). The composite seed was modeled as a cylinder. The distance between the two composite seeds in Figure S25b (Supporting Information), as well as the distances between the seeds in the coronal plane and sagittal plane in Figure S25c (Supporting Information), was defined as D. Their influences on the temperature distribution were investigated.

Three Pennes bio‐heat transfer equations for simulating the temperature changes in composite seed, tumor, and healthy tissue were Equations ((2), (3), (4)).^[^ ^45^ ^]^ The Pennes bio‐heat transfer equations were solved by COMSOL.

(2) $$\rho_{S}c_{S}\frac{\partial T}{\partial t}=\frac{k_{S}}{r^{2}}\frac{\partial \left(r^{2}\frac{\partial T}{\partial r}\right)}{\partial r}+P\;\;\;\;\;\;\;\;\;\;\;\;\;\;\left(\mathrm{Composite}\;\mathrm{seed}\right)$$

(3) $$\rho_{T}c_{T}\frac{\partial T}{\partial t}=\frac{k_{T}}{r^{2}}\frac{\partial \left(r^{2}\frac{\partial T}{\partial r}\right)}{\partial r}+Q_{\mathrm{mT}}+Q_{\mathrm{bT}}\;\;\;\;\;\;\;\;\;\;\;\;\;\;\left(\mathrm{Tumor}\right)$$

(4) $$\rho_{L}c_{L}\frac{\partial T}{\partial t}=\frac{k_{L}}{r^{2}}\frac{\partial \left(r^{2}\frac{\partial T}{\partial r}\right)}{\partial r}+Q_{\mathrm{mL}}+Q_{\mathrm{bL}}\;\;\;\;\;\;\left(\mathrm{Liver}\right)$$

The initial condition was Equation (5).

(5) $$T=37^{\circ }C$$

At the interface of the tumor and healthy tissue, the temperature and heat flow was continuous. And the outer boundary condition was Equation (6).

(6) $${\left.T\right|}_{outer\;surface\;of\;liver}=37^{\circ }C$$

where, *T* was the temperature (°C); *r* was the radius from the center (m); ρ was the density (kg/m^3^); *c* was the specific heat capacity (J/(kg·°C)); *k* was the thermal conductivity (W/(m·°C)); *Q*_m_ was the power density of metabolic heat generation (W/m^3^); *Q*_b_ was the power density of heat dissipation by blood perfusion effect (W/m^3^); *P* was the power density of composite seed, *i*.*e*., the power dissipation of composite seed per unit of volume (W/m^3^) ^42^; the subscript S represents composite seed, T represents the tumor, L represents the healthy liver tissue. The ρ, *c*, and *k* of the composite seed were Equation (78).^[^ ^43^, ^44^ ^]^

(7) $$\left\{\begin{array}{cc} & \rho_{S}=\phi_{\mathrm{MNP}}\rho_{\mathrm{MNP}}+\phi_{\mathrm{Ag}}\rho_{\mathrm{Ag}}+\phi_{\mathrm{Ti}}\rho_{\mathrm{Ti}}+\phi_{\mathrm{Air}}\rho_{\mathrm{Air}}\\ & c_{S}=\phi_{\mathrm{MNP}}c_{\mathrm{MNP}}+\phi_{\mathrm{Ag}}c_{\mathrm{Ag}}+\phi_{\mathrm{Ti}}c_{\mathrm{Ti}}+\phi_{\mathrm{Air}}c_{\mathrm{Air}}\\ & \frac{1}{k_{S}}=\frac{\phi_{\mathrm{MNP}}}{k_{\mathrm{MNP}}}+\frac{\phi_{\mathrm{Ag}}}{k_{\mathrm{Ag}}}+\frac{\phi_{\mathrm{Ti}}}{k_{\mathrm{Ti}}}+\frac{\phi_{\mathrm{Air}}}{k_{\mathrm{Air}}} \end{array}\right.$$

where, the subscript MNP represents MNPs, Ag represents silver rod, Ti represents titanium shell, Air represents air gaps in the composite seed; ϕ was the volume fraction of each component in the seed.

The *Q*_m_ and *Q*_b_ rely on the body temperature change. The *Q*_m_ was Equation (8).^[^ ^46^ ^]^

(8) $$Q_{m}=Q_{m0}\left[1+0.1\left(T-37\right)\right]$$

where, *Q*_m0_ was the *Q*_m_ when tissue was at 37°C (W/m^3^).

The *Q*_b_ was Equation (9).^[^ ^47^ ^]^

(9) $$Q_{b}=w_{b}\rho_{b}c_{b}\times \left(T_{\alpha }-T\right)$$

where, the subscript b represents blood; *w*_b_ was the blood perfusion rate (1/s); *T*_α_ was the temperature of arterial blood (°C), here assumed as 37°C.

The physical parameters of tissues and composite seed were shown in **Table** **1**.

**Table 1:** Physical parameters of tissues and composite seed.

	*ρ*	*c*	*k*	*w*_b_	*Q*_m0_	ϕ
	kg/m^3^	J/(kg·°C)	W/(m·°C)	1/s	W/m^3^	%
Tumor^[^ ^42^ ^]^	1060	3540	0.52	0.000833	5790	N/A
Blood^[^ ^48^ ^]^	1050	3617	N/A	N/A	N/A	N/A
Liver^[^ ^48^ ^]^	1079	3540	0.52	0.0155	10682	N/A
MNPs^[^ ^42^ ^]^	5180	670	40	N/A	N/A	16.56
Silver^[^ ^49^ ^]^	10490	235	429	N/A	N/A	28.88
Titanium^[^ ^49^ ^]^	4500	523	21.90	N/A	N/A	40.90
Air^[^ ^49^ ^]^	1.184	1005	0.0262	N/A	N/A	13.66

The *P* was calculated as Equation (10).^[^ ^50^ ^]^

(10) $$P=SLP\times \rho_{S}$$

where, *SLP* was the specific loss power of the composite seed (W/kg).

The *SLP*‐*T* data of the composite seed was fitted with Gaussian curves to provide the heat source in simulations with continuous coupling relationships. The fitting equation was Form 11.

(11) $$SLP\left(T\right)=a\times e^{-{\left(\frac{T-b}{c}\right)}^{2}}+d$$

where, *a*, *b*, *c*, and *d* were the fitting parameters, which were 0.972, 32.002, 19.012, and 0.782, respectively. *R* ^2^ was 0.934.

### Subcutaneous Hyperthermia Simulation

The model was adjusted for subcutaneuos experiment, as shown in Figure S26 (Supporting Information). An ellipsoidal tumor (diameter: 10 mm, 7 mm, 4 mm) was modeled with 1/5 minor axis embedded in healthy muscle tissue, mimicking a superficial tumor (skin neglected). A composite seed was implanted into the tumor center. The air was beyond the tissue. The parameters of the muscle were 1,090 kg/m3 of density, 3421 J/(kg·°C) of heat capacity, 0.49 W/(m·°C) of thermal conductivity, 0.000643 1/s of blood perfusion rate, and 988 W/m^3^ of metabolic heat generation power density.^[^ ^48^ ^]^ A natural convection condition of air was applied on the upper surface of the tumor and tissue.

### Cell Line Culture

Hepa l‐6 murine hepatoma cells, N1S1 rat hepatoma cells, RM‐1 murine PCa cells, and TRAMP‐C2 murine PCa cells were purchased from the American Type Culture Collection (ATCC, USA). Hepa l‐6 and N1S1 were cultured in a DMEM medium (Gibco, Thermo Fisher Scientific, Waltham, MA, USA) containing 10% fetal bovine serum (FBS; Gibco), 1% penicillin G, and streptomycin sodium (Gibco) in 95% humidified air at 37°C and 5% CO_2_. RM‐1 and TRAMP‐C2 cells were cultured in an RPMI 1640 medium (Gibco, Thermo Fisher Scientific, Waltham, MA, USA) containing 10% fetal bovine serum (FBS; Gibco), 1% penicillin G, and streptomycin sodium (Gibco) in 95% humidified air at 37°C and 5% CO_2_.

### Animal Models

The use of laboratory mice was approved by the IEC for Clinical Research of Zhongda Hospital, Affiliated to Southeast University (Approval No. 20190403001).

### Animal Models—Liver Cancer and Pca Subcutaneous Tumor Model

C57BL/6 mice (only male for PCa) aged 4–6 weeks were purchased from the Beijing Vital River Laboratory Animal Technology Co., Ltd. and raised under the standard conditions at the Animal Center of Southeast University. Mice were acclimated in a laboratory environment for 2–3 weeks until 6–8 weeks of age. RM‐1 (2 × 10^6^) cells, TRAMP‐C2 (2 × 10^6^) cells, or Hepa l‐6 (4 × 10^6^) cells were injected subcutaneously into the right inguinal region of each mouse.

### Animal Models—Liver Cancer Orthotopic Tumor Model

7‐week‐old SD rats were purchased from Beijing Vital River Laboratory Animal Technology Co., Ltd, and raised under standard conditions at the Animal Center of Southeast University. Rats were acclimated in a laboratory environment for 2–3 weeks until 9–10 weeks of age. The rats were anesthetized with inhalation anesthesia and placed in a supine position. The abdomen was disinfected with iodine, and a midline incision (≈2–3 cm) was made in the upper abdomen to expose the liver. N1S1 cells (1 × 10^6^) were injected into the desired liver lobe. The injection site was gently pressed with sterile gauze for 1–2 min, and the liver was carefully returned to its original position. The incision was closed with absorbable sutures. MRI was performed every three days to confirm tumor formation and monitor tumor growth.

### Animal Models—Treatments and Measurements for Subcutaneous Tumors and N1S1 Orthotopic Tumors

On day 0 (14 days after tumor bearing), when the diameter of the tumor reached ≈6 mm, blank seeds, ^125^I seeds, and ^125^I@MH seeds were implanted into the subcutaneous tumor. On the second day after seed implantation, the ^125^I@MH group was heated in a magnetic field for 30 min (magnetic field conditions: H = 400 ± 5 Oe, f = 100 ± 5 kHz), during which real‐time temperature monitoring was performed using an infrared thermometer and a fiber optic thermometer. Tumor volume was then evaluated on day 1, 3, 6, and 10 after injection with length × width^2^ × 0.5. The mice were sacrificed, and tumors were harvested on day 10 for weighing, flow cytometry, and mIF staining.

### KI67 Assay

Take an appropriate amount of fixed tissue sample and process it into routine paraffin sections. The sample was deparaffinized to water using xylene and subjected to antigen retrieval, commonly performed using high‐temperature and high‐pressure methods or enzymatic digestion. Subsequently, wash the sample with PBS buffer and block endogenous peroxidase activity and non‐specific binding sites. Add appropriately diluted KI67 primary antibody (GB111141, ServiceBio, 1:1000), incubating overnight at 4°C or for 1–2 h at room temperature. After washing, add the secondary antibody (GB23301, ServiceBio, 1:200), incubate further, and wash again. Use DAB for color development, and terminate the reaction promptly based on the intensity of the staining. Counterstain the nuclei with hematoxylin, dehydrate, clear, and mount the sections. Observe under a microscope and record the number and distribution of KI67‐positive cells.

### TUNEL Assay

Take fixed tissue sections and treat them with proteinase K to enhance membrane permeability. Then wash the sections with PBS to remove residual enzymes. Prepare the TUNEL reaction mixture according to the kit instructions, which includes TdT (terminal deoxynucleotidyl transferase) and fluorescently labeled dUTP. Apply the reaction mixture to the sample surface and incubate at 37°C for 1 h to allow the labeled dUTP to bind to DNA strand breaks. After incubation, wash thoroughly with PBS to remove nonspecific bindings. Additionally, use a blue dye such as DAPI to counterstain the nuclei for contrast. Finally, observe the samples under a fluorescence microscope. Apoptotic cells with fragmented DNA ends will exhibit green fluorescence, while the nuclei will show blue fluorescence, allowing for the clear identification of cell positions and the degree of apoptosis.

### In Vitro Models for ^125^I Irradiation and Heating

In vitro models were applied to evaluate whether combined therapy yields superior therapeutic outcomes compared to radiotherapy alone. According to the previous method,^[^ ^51^ ^]^ an in vitro cellular ^125^I irradiation model was established. The irradiation setup consisted of a radiation source carrier platform and a cell culture dish, forming a complete brachytherapy experimental system. The radiation source carrier platform was a 35‐mm diameter polystyrene culture dish that was filled with paraffin wax, with one ^125^I seed embedded at the center and eight additional seeds symmetrically arranged along the periphery, serving as the radiation source. Dosimetric calculations indicated that a total exposure time of 92 h was required to deliver a cumulative radiation dose of 4 Gy. A separate 35‐mm culture dish containing a monolayer of adherent cells was placed directly above the radiation source at a fixed vertical distance, ensuring uniform and controlled irradiation. The assembled setup was first incubated in a 43°C water bath for 30 min to simulate hyperthermic preconditioning, and subsequently transferred to a 37°C incubator for continued cell culture and irradiation.

### Live/Dead Cell Viability Assays

Cell viability was assessed using Live/dead cell viability assay via Calcein/PI Cell Viability and Cytotoxicity Reagent (Beyotime Biotechnology). Calcein/PI dyes were prepared according to the instructions and added to the cell suspension after experimental treatment. After incubating for 10 min, staining results were assessed with a fluorescence microscope.

### DNA Double‐Strand Break Detection

After experimental treatment, the cells were washed, fixed, permeabilized, and blocked, followed by the addition of γ‐H2AX rabbit primary antibody (Beyotime, Biotechnology) and incubation at 4 °C overnight. Nuclear staining solution (DAPI) was then added, and the cells were stained at room temperature for ≈5 min. The cells were then mounted on slides and observed using a fluorescence microscope.

### CRT Expression and Localization Observation

After experimental treatment, the cells were washed, fixed, permeabilized, and blocked. The cells were then incubated with a primary antibody against calreticulin (*Cat*: Ab2907, *Company*: Abcam, 1:200) at 4 °C overnight. Afterward, the cells were incubated with a fluorescently labeled secondary antibody for 1 h, followed by DAPI staining to label the nuclei. The cells were then mounted on slides and observed under a confocal microscope to analyze the expression and localization of calreticulin.

### Multiplexed Immunofluorescence Staining (mIF)

The mIF staining protocol was performed using a Sive‐color multi‐labeled immunofluorescence staining kit (abs50014, Absin) according to the instructions. Briefly, sections were deparaffinized, rehydrated, subjected to antigen retrieval (Tris‐EDTA antigen retrieval solution, G1203, Servicebio; 10 min), and then blocked with PBS containing 10% goat serum for 20 min at room temperature, followed by incubation with the primary antibody overnight at 4 °C. After washing, sections were incubated with a secondary antibody for 1 h at room temperature, and tyramide signal amplification (TSA) was performed. Then, antigen retrieval was conducted again for the staining for the second primary antibody. After all primary antibodies and corresponding secondary antibodies were stained, sections were incubated with DAPI for 10 min, and finally sealed with an anti‐fluorescence quenching sealing agent. The primary antibodies included Epcam (*Cat*: GB11274, *Company*: CiteAB, 1:2,000), Cxcl2 (16325‐1‐AP, Proteintech, 1:200), Csf3r (orb1676847, Biorbyt, 1:2,000), Cxcr2 (CSB‐PA009567, CUSABio, 1:200), Cd274/Pd‐l1 (28076‐1‐AP, Proteintech, 1:200), Cd3 (17‐0031‐82, Thermo Fisher, 1:200), Cd8 (11‐0081‐82, Thermo Fisher, 1:200), Ifn‐γ (12‐7311‐82, Thermo Fisher, 1:200), Pd‐1 (66220‐1‐lg, Proteintech, 1:200), F4/80 (123116, 1:100, BioLegend), Ccr2 (MAB55382, R&D Systems, 1:200), Apoe (66830‐1‐lg, Proteintech, 1:200). Cells were labeled with antibodies and then stained with DAPI. Images were acquired by laser scanning confocal microscopy (LSM700, Zeiss) and processed by *Zen* software (v.2.3, Zeiss). *ImageJ* software (v.1.53t) was used to obtain the fluorescence intensity represented by the mean grey value through setting a threshold for signal capturing.

### Flow Cytometry Assay

The fresh tumor tissues were washed (Hanks Balanced Salt Solution), minced, digested (GEXSCOPE Tissue Dissociation Solution), centrifuged, and resuspended to obtain single‐cell suspensions. Cells were stained with antibodies including Fixable Viability Dye (*Dye*: APC‐CY7, *Cat*: 565388, *Company*: BD), Cd45 (PerCP‐Cy5.5, 550994, BD; BV510, 103137, BD; FITC, 553079, BD; APC, 147708, BioLegend), Csf3r (APC, orb1676847, Biorbyt), Cd274 (PE, 558091, BD), Cd3 (FITC, 100203, BioLegend; PE, 100206, BioLegend), Pd‐1 (PE, 135215, BioLegend), Ifn‐γ (BV786, 563773, BD), Cd68 (APC, 137008, BioLegend), Ccr2 (PE, 150610, BioLegend), F4/80 (BV421, 565411, BD), Cd206 (APC, 141708, BioLegend) and Cd8a (PerCP, 100732, BioLegend). Staining was carried out following the Intracellular Flow Cytometry guideline from Invitrogen. Briefly, cells were first stained for surface protein antibodies, and then fixed, permeabilized, and washed for staining of intracellular protein antibodies. Flow cytometry data were obtained using the CytoFLEX Cytometer (Beckman Coulter, Villepinte, France) and analyzed with *FlowJo* software (v.10.8.1, Celeza). The flow cytometry panels were as follows:

(a) Cd274^+^ Neutrophils: Singlets → Live cells → Cd45^+^ → Csf3r^+^ & Cd274^+^

(b) Effector T cells: Singlets → Live cells → Cd45^+^ → Cd3^+^ & Ifn‐γ^+^

(c) Exhausted T cells: Singlets → Live cells → Cd45^+^ → Cd3^+^ & Pd1^+^

(d) Ccr2^+^ Macrophages: Singlets → Live cells → Cd45^+^ → Cd68^+^ & Ccr2^+^

(e) Cd206^+^ Macrophages: Singlets → Live cells → Cd45^+^ → F4/80^+^ & Cd206^+^

(f) Cd8^+^ T cells: Singlets → Live cells → Cd45^+^ → Cd3^+^ & Cd8a^+^

### Interpreting of scRNA‐seq Data

Raw gene expression matrices were generated for each sample by the Cell Ranger pipeline coupled with the mouse reference version GRCm38. The output filtered gene expression matrices were analyzed by R software with the *Seurat* (v.5.0.1) program.^[^ ^52^ ^]^ In brief, genes expressed at a proportion > 0.1% of the data and cells with >200 genes detected were selected for further analyses. Low‐quality cells were removed if they met the following criteria: 1) < 200 genes; 2) > 30000 genes; 3) > 10% UMIs derived from the mitochondrial genome.

### Interpreting of scRNA‐seq Data—Batch Effect Removal and Clustering

Gene expressions were first normalized with *LogNormalize* method. The top 2,000 variable genes were identified with *FindVariableFeatures* function and scaled and regressed out for the expression percentage of mitochondrial genes with *ScaleData* function, which were used for principal component analysis (PCA) via *RunPCA* function. The first 20 dimensions were used for batch effect removal and clustering. The Batch effect was assessed using *RunHarmony* function of R package *Harmony* (v.1.2.0)^[^ ^53^ ^]^ for the development of a batch‐corrected Harmony dimensionality reduction. Uniform Manifold Approximation and Projection (UMAP) dimensionality reduction on the Harmony dimensions to obtain 2D representations for data visualization. We then computed the shared nearest neighbors (SNNs) with *FindNeighbors* and identified clusters with *FindClusters* using Harmony dimensions. Resolutions used for clustering of all cells, myeloid cells, and lymphoid cells were 2.5, 0.6, and 0.6, respectively.

### Interpreting of scRNA‐seq Data—Cell Clusters Annotation

The cluster‐specific marker genes were applied as reference, which were identified with the *Wilcoxon* test implemented in *FindAllMarkers* function. Clusters were then classified and annotated based on the expression of canonical markers of particular cell types.

### Interpreting of scRNA‐seq Data—Deciphering the Intro‐Tumoral Transcriptional Programs

To investigate potential intra‐tumoral signatures of PCa, non‐negative matrix factorization (NMF, implemented in the *cNMF* package v.1.4 in Python) was performed on malignant epithelia. NMF could extract programs with biological significance from the combinations of cellular transcriptional modules.^[^ ^54^ ^]^ The top 3,000 highly variable genes were extracted, and their expressions were scaled, and replaced all negative expression values were replaced with zero. The top co‐expressed gene modules in each sample were profiled, from which the 50 genes with the highest weight were extracted to define intra‐tumoral transcriptional programs. Between 3 and 20 transcriptional programs were obtained in each sample, and only the retained ones with a standard deviation larger than 0.2 were considered. Further, clustering underlying the pairwise Jaccard index was performed to identify programs shared among different samples, which were named meta‐programs (MPs). Genes with expression percentages of more than 30% among all malignant epithelia were defined as hub genes for MPs.

### Interpreting of scRNA‐seq Data—DEGs Identification and Functional Enrichment

Differential gene expression testing was performed using the FindMarkers function in *Seurat* with the parameter logfc.threshold = 0.25, min.pct = 0.1, and other parameters by default, and the Benjamini‐Hochberg method was used to estimate the adjusted *P* value. DEGs were filtered using a maximum adjusted *P* value of 0.05. DEGs were used for subsequent GSEA, performed using the clusterProfiler package in R.^[^ ^55^ ^]^ Gene sets queried included Gene Ontology collections available through the Molecular Signatures Database (MSigDB).

### Statistical Analysis

All statistical analyses were carried out using *R* (v.4.0.5) and *python* (v.3.7.12). The gene expression data and human specimens‐derived experimental data often do not follow a normal distribution, so we employed non‐parametric tests for statistical tests. Specifically, the Wilcoxon rank‐sum test was used to compare differences between two groups, while the Kruskal‐Wallis test was used to compare differences among multiple groups. For the in vitro and in vivo experimental data, the distribution was assumed to be normal, but this was not formally tested. The *t*‐test and one‐way ANOVA test were used for group comparisons. *χ^2^* tests were used as statistical tests for categorical variables. All tests were two‐sided. No animal or data point was excluded from the statistical analyses.

## Conflict of Interest

The authors declare no conflict of interest.

## Author Contributions

B.X., Y.M.W., Y.F.C., L.D., S.W.D., and S.R.C. contributed equally to this work. The conceptualization of the study was led by G.T. Methodology was developed by B.X., W.Z., Y.M.W., Y.C., and C.W. Formal analysis was conducted by Y.C. and S.C., while the investigation was carried out by Y.M.W., L.D., S.D., R.Y., W.H.Z., Y.J.W., W.M., F.S., Z.J., J.C., E.Q., Y.Q.W., and P.Z. Resources were provided by G.T., J.X., W.Z., S.D., and B.C. Project administration was undertaken by B.X. and J.L., with supervision provided by G.T., B.X., J.X., W.Z., and J.L. Validation was performed by Y.M.W. Funding acquisition was the responsibility of G.T. and B.X. The original draft of the manuscript was prepared by Y.M.W. and Y.C., and the writing review and editing were conducted by G.T., B.X., J.L., and S.D.

## Supporting information



Supporting Information

Supporting Figures

## Data Availability

The data generated from this study were deposited on the CNGB Sequence Archive (CNSA) under the project CNP0006802. The scRNA‐seq data were accessible with accession number CSE0000534.

## References

[1] K. Tanderup , C. Ménard , C. Polgar , J. C. Lindegaard , C. Kirisits , R. Pötter , Adv. Drug Deliv. Rev. 2017, 109, 15. 27637454 10.1016/j.addr.2016.09.002

[2] Q. Jin , C. Lin , X. Zhu , Y. Cao , C. Guo , L. Wang , Radiat. Oncol. 2020, 15, 238. 33059701 10.1186/s13014-020-01682-5 PMC7559445

[3] H. E. Barker , J. T. Paget , A. A. Khan , K. J. Harrington , Nat. Rev. Cancer 2015, 15, 409. 26105538 10.1038/nrc3958 PMC4896389

[4] M. McLaughlin , E. C. Patin , M. Pedersen , A. Wilkins , M. T. Dillon , A. A. Melcher , K. J. Harrington , Nat. Rev. Cancer 2020, 20, 203. 32161398 10.1038/s41568-020-0246-1

[5] X. Yao , S. Lu , C. Feng , R. Suo , H. Li , Y. Zhang , Q. Chen , J. Lu , B. Wu , J. Guo , Biomaterials 2022, 289, 121801. 36137416 10.1016/j.biomaterials.2022.121801

[6] Z. Jin , Y. Du , Z. Li , Y. Jiang , J. Chen , Y. Liu , Endoscopy 2008, 40, 314. 18283622 10.1055/s-2007-995476

[7] Z. Huang , W. Yao , Z. Zhong , G. Yang , J. Liu , H. Gu , J. Huang , Heliyon 2024, 10, 24666. 10.1016/j.heliyon.2024.e24666 PMC10828072 38298696

[8] T. S. Quang , L. W. Brady , Int. J. Radiat. Oncol. Biol. Phys. 2004, 58, 972.14967458

[9] P. Vaupel , H. Piazena , M. Notter , A. R. Thomsen , A.‐L. Grosu , F. Scholkmann , A. G. Pockley , G. Multhoff , Cancers 2023, 15, 394. 36900190 10.3390/cancers15051394 PMC10000497

[10] M. W. Dewhirst , B. L. Viglianti , M. Lora‐Michiels , M. Hanson , P. J. Hoopes , Int. J. Hyperthermia 2003, 19, 267. 12745972 10.1080/0265673031000119006

[11] A. Farzin , S. A. Etesami , J. Quint , A. Memic , A. Tamayol , Adv. Healthc. Mater. 2020, 9, 1901058. 10.1002/adhm.201901058 PMC7482193 32196144

[12] J. Beik , Z. Abed , F. S. Ghoreishi , S. Hosseini‐Nami , S. Mehrzadi , A. Shakeri‐Zadeh , S. K. Kamrava , J. Controlled Release 2016, 235, 205. 10.1016/j.jconrel.2016.05.062 27264551

[13] N. R. Datta , S. G. Ordóñez , U. S. Gaipl , M. M. Paulides , H. Crezee , J. Gellermann , D. Marder , E. Puric , S. Bodis , Cancer Treat. Rev. 2015, 41, 742. 26051911 10.1016/j.ctrv.2015.05.009

[14] T. M. Zagar , J. R. Oleson , Z. Vujaskovic , M. W. Dewhirst , O. I. Craciunescu , K. L. Blackwell , L. R. Prosnitz , E. L. Jones , Inte. J. Hyperthermia 2010, 26, 612. 10.3109/02656736.2010.487194 PMC2956421 20849256

[15] J. Overgaard , Radiother. Oncol. 2013, 109, 185. 24314332 10.1016/j.radonc.2013.11.004

[16] R. D. Issels , L. H. Lindner , J. Verweij , R. Wessalowski , P. Reichardt , P. Wust , P. Ghadjar , P. Hohenberger , M. Angele , C. Salat , Z. Vujaskovic , S. Daugaard , O. Mella , U. Mansmann , H. R. Dürr , T. Knösel , S. Abdel‐Rahman , M. Schmidt , W. Hiddemann , K.‐W. Jauch , C. Belka , A. Gronchi , JAMA Oncol. 2018, 4, 483. 29450452 10.1001/jamaoncol.2017.4996 PMC5885262

[17] R. D. Issels , Eur. J. Cancer 2008, 44, 2546. 18789678 10.1016/j.ejca.2008.07.038

[18] N. R. Datta , H. P. Kok , H. Crezee , U. S. Gaipl , S. Bodis , Front. Oncol. 2020, 10, 819. 32596144 10.3389/fonc.2020.00819 PMC7303270

[19] F. Qi , Q. Bao , P. Hu , Y. Guo , Y. Yan , X. Yao , J. Shi , Biomaterials 2024, 307, 122514. 38428093 10.1016/j.biomaterials.2024.122514

[20] X. Yu , R. Yang , C. Wu , W. Zhang , J. Alloys Compd. 2020, 830, 154724.

[21] X. Yu , M. Mostafezur Rahman , R. Yang , C. Wu , A. Bouyahya , W. Zhang , J. Magn. Magn. Mater. 2024, 591, 171724.

[22] M. A. Giese , L. E. Hind , A. Huttenlocher , Blood 2019, 133, 2159. 30898857 10.1182/blood-2018-11-844548 PMC6524564

[23] B. B. Antuamwine , R. Bosnjakovic , F. Hofmann‐Vega , X. Wang , T. Theodosiou , I. Iliopoulos , S. Brandau , Immunol. Rev. 2023, 314, 250. 36504274 10.1111/imr.13176

[24] M. T. Dillon , K. F. Bergerhoff , M. Pedersen , H. Whittock , E. Crespo‐Rodriguez , E. C. Patin , A. Pearson , H. G. Smith , J. T. E. Paget , R. R. Patel , S. Foo , G. Bozhanova , C. Ragulan , E. Fontana , K. Desai , A. C. Wilkins , A. Sadanandam , A. Melcher , M. McLaughlin , K. J. Harrington , Clin. Cancer Res. 2019, 25, 3392. 30770349 10.1158/1078-0432.CCR-18-1821 PMC6551222

[25] A. Obradovic , N. Chowdhury , S. M. Haake , C. Ager , V. Wang , L. Vlahos , X. V. Guo , D. H. Aggen , W. K. Rathmell , E. Jonasch , J. E. Johnson , M. Roth , K. E. Beckermann , B. I. Rini , J. McKiernan , A. Califano , C. G. Drake , Cell 2021, 184, 2988. 34019793 10.1016/j.cell.2021.04.038 PMC8479759

[26] M. Revel , C. Sautès‐Fridman , W. H. Fridman , L. T. Roumenina , Trends Cancer 2022, 8, 517. 35288093 10.1016/j.trecan.2022.02.006

[27] R. Bill , P. Wirapati , M. Messemaker , W. Roh , B. Zitti , F. Duval , M. Kiss , J. C. Park , T. M. Saal , J. Hoelzl , D. Tarussio , F. Benedetti , S. Tissot , L. Kandalaft , M. Varrone , G. Ciriello , T. A. McKee , Y. Monnier , M. Mermod , E. M. Blaum , I. Gushterova , A. L. K. Gonye , N. Hacohen , G. Getz , T. R. Mempel , A. M. Klein , R. Weissleder , W. C. Faquin , P. M. Sadow , D. Lin , et al., Science 2023, 381, 515. 37535729 10.1126/science.ade2292 PMC10755760

[28] D. Dangaj , M. Bruand , A. J. Grimm , C. Ronet , D. Barras , P. A. Duttagupta , E. Lanitis , J. Duraiswamy , J. L. Tanyi , F. Benencia , J. Conejo‐Garcia , H. R. Ramay , K. T. Montone , D. J. Powell , P. A. Gimotty , A. Facciabene , D. G. Jackson , J. S. Weber , S. J. Rodig , S. F. Hodi , L. E. Kandalaft , M. Irving , L. Zhang , P. Foukas , S. Rusakiewicz , M. Delorenzi , G. Coukos , Cancer Cell 2019, 35, 885. 31185212 10.1016/j.ccell.2019.05.004 PMC6961655

[29] Q. Gu , L. Zhu , Bioengineering (Basel) 2024, 11, 900. 39329642 10.3390/bioengineering11090900 PMC11428587

[30] C. M. van Leeuwen , A. L. Oei , K. W. T. K. Chin , J. Crezee , A. Bel , A. M. Westermann , M. R. Buist , N. A. P. Franken , L. J. A. Stalpers , H. P. Kok , Radiat. Oncol. 2017, 12, 75. 28449703 10.1186/s13014-017-0813-0 PMC5408439

[31] W. Zhang , X. Yu , H. Li , D. Dong , X. Zuo , C. Wu , J. Magn. Magn. Mater. 2019, 489, 165382.

[32] T.‐T. Wang , Y.‐L. Zhao , L.‐S. Peng , N. Chen , W. Chen , Y.‐P. Lv , F.‐Y. Mao , J.‐Y. Zhang , P. Cheng , Y.‐S. Teng , X.‐L. Fu , P.‐W. Yu , G. Guo , P. Luo , Y. Zhuang , Q.‐M. Zou , Gut 2017, 66, 1900. 28274999 10.1136/gutjnl-2016-313075 PMC5739867

[33] W. Jing , G. Wang , Z. Cui , X. Li , S. Zeng , X. Jiang , W. Li , B. Han , N. Xing , Y. Zhao , S. Chen , B. Shi , Proc. Natl. Acad. Sci. USA 2024, 121, 2312855121. 10.1073/pnas.2312855121 PMC11098120 38713626

[34] D. Sun , L. Tan , Y. Chen , Q. Yuan , K. Jiang , Y. Liu , Y. Xue , J. Zhang , X. Cao , M. Xu , Y. Luo , Z. Xu , Z. Xu , W. Xu , M. Shen , J. Experim. Clin. Cancer Res. 2024, 43, 202. 10.1186/s13046-024-03122-8 PMC11264977 39034411

[35] R. Xue , Q. Zhang , Q. Cao , R. Kong , X. Xiang , H. Liu , M. Feng , F. Wang , J. Cheng , Z. Li , Q. Zhan , M. Deng , J. Zhu , Z. Zhang , N. Zhang , Nature 2022, 612, 141. 36352227 10.1038/s41586-022-05400-x

[36] H. Deng , A. Kan , N. Lyu , M. He , X. Huang , S. Qiao , S. Li , W. Lu , Q. Xie , H. Chen , J. Lai , Q. Chen , X. Jiang , S. Liu , Z. Zhang , M. Zhao , J. Immun. Cancer 2021, 9, 002305. 10.1136/jitc-2020-002305 PMC8231064 34168004

[37] P. Xie , M. Yu , B. Zhang , Q. Yu , Y. Zhao , M. Wu , L. Jin , J. Yan , B. Zhou , S. Liu , X. Li , C. Zhou , X. Zhu , C. Huang , Y. Xu , Y. Xiao , J. Zhou , J. Fan , M.‐C. Hung , Q. Ye , L. Guo , H. Li , J. Hepatology 2024, 81, 93. 10.1016/j.jhep.2024.02.009 38403027

[38] E. Nolan , V. L. Bridgeman , L. Ombrato , A. Karoutas , N. Rabas , C. A. N. Sewnath , M. Vasquez , F. S. Rodrigues , S. Horswell , P. Faull , R. Carter , I. Malanchi , Nat. Cancer 2022, 3, 173. 35221334 10.1038/s43018-022-00336-7 PMC7612918

[39] C. Yang , H. Geng , X. Yang , S. Ji , Z. Liu , H. Feng , Q. Li , T. Zhang , S. Zhang , X. Ma , C. Zhu , N. Xu , Y. Xia , Y. Li , H. Wang , C. Yu , S. Du , B. Miao , L. Xu , H. Wang , Y. Cao , B. Li , L. Zhu , X. Tang , H. Zhang , C. Zhu , Z. Huang , C. Leng , H. Hu , X. Chen , et al., Cancer Cell 2024, 42, 2064. 39515328 10.1016/j.ccell.2024.10.008

[40] X. Kan , G. Zhou , F. Zhang , H. Ji , D. S. Shin , W. Monsky , C. Zheng , X. Yang , J. Immunotherapy of Cancer 2022, 005619, 10. 10.1136/jitc-2022-005619 PMC9717415 36450380

[41] W. Zhang , X. Jin , H. Li , R. R. Zhang , C. W. Wu , Carbohydr. Polym. 2018, 186, 82. 29456012 10.1016/j.carbpol.2018.01.008

[42] Y. Tang , R. C. C. Flesch , T. Jin , J. Appl. Phys. 2017, 122, 034702.

[43] Q. Wang , Z. S. Deng , J. Liu , J. Nanopart. Res. 2012, 14, 974.

[44] I. Astefanoaei , I. Dumitru , H. Chiriac , A. Stancu , IEEE Trans. Magn. 2016, 52, 1.

[45] M. Ebrahimi , J. Magn. Magn. Mater. 2016, 416, 134.

[46] P. K. Gupta , J. Singh , K. N. Rai , J. Thermal Biol. 2010, 35, 295.

[47] H. H. Pennes , J. Appl. Physiol. 1998, 85, 5. 9714612 10.1152/jappl.1998.85.1.5

[48] C. Baumgartner , P. A. Hasgall , F. Di Gennaro , E. Neufeld , B. Lloyd , M. C. Gosselin , D. Payne , A. Klingenböck , N. Kuster , “IT'IS Database for thermal and electromagnetic parameters of biological tissues,” Version 5.0, August 21, 2025. itis.swiss/database.

[49] W. M. Haynes , CRC Handbook of Chemistry and Physics, CRC Press, United States, 2016.

[50] I. Raouf , S. Khalid , A. Khan , J. Lee , H. S. Kim , M. H. Kim , J Therm Biol 2020, 91, 102644. 32716885 10.1016/j.jtherbio.2020.102644 PMC7410490

[51] E. G. Aird , M. Folkard , C. R. Mayes , P. J. Bownes , J. M. Lawson , M. C. Joiner , Br. J. Radiol. 2001, 74, 56. 11227778 10.1259/bjr.74.877.740056

[52] Y. Hao , S. Hao , E. Andersen‐Nissen , W. M. Mauck , S. Zheng , A. Butler , M. J. Lee , A. J. Wilk , C. Darby , M. Zager , P. Hoffman , M. Stoeckius , E. Papalexi , E. P. Mimitou , J. Jain , A. Srivastava , T. Stuart , L. M. Fleming , B. Yeung , A. J. Rogers , J. M. McElrath , C. A. Blish , R. Gottardo , P. Smibert , R. Satija , Cell 2021, 184, 3573. 34062119 10.1016/j.cell.2021.04.048 PMC8238499

[53] I. Korsunsky , N. Millard , J. Fan , K. Slowikowski , F. Zhang , K. Wei , Y. Baglaenko , M. Brenner , P.‐R. Loh , S. Raychaudhuri , Nat. Methods 2019, 16, 1289. 31740819 10.1038/s41592-019-0619-0 PMC6884693

[54] BRAIN Initiative Cell Census Network (BICCN) , Nature 2021, 598, 86.34616075

[55] G. Yu , L. G. Wang , Y. Han , Q. Y. He , Omics ,J. Integr. Biol. 2012, 16, 284. 10.1089/omi.2011.0118 PMC3339379 22455463

