# Supplementary material for: A Novel Self‐Regulated, Non‐Directional Magnetic Thermos‐Brachytherapy 125I Seed Enhances Anticancer Efficacy by Rescuing Immune Escape
Source: Adv Sci (Weinh). 2025 Aug 29;12(43):e08091. doi: 10.1002/advs.202508091 (PMC12631875; doi:10.1002/advs.202508091)
Supplement: Supplementary file 1 — Supporting Information [file ADVS-12-e08091-s001.docx]

**Figure S1.** MNPS transmission electron microscopy-based size distribution image.

**
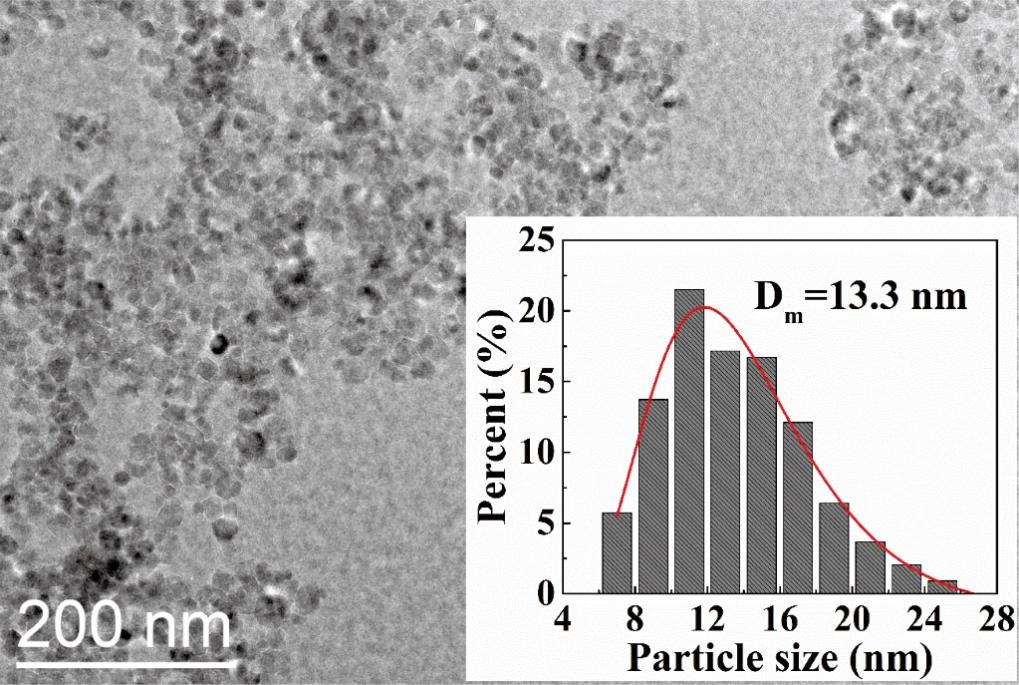
**

**Figure S2.** High-resolution transmission electron microscopy image of MNPs.

**
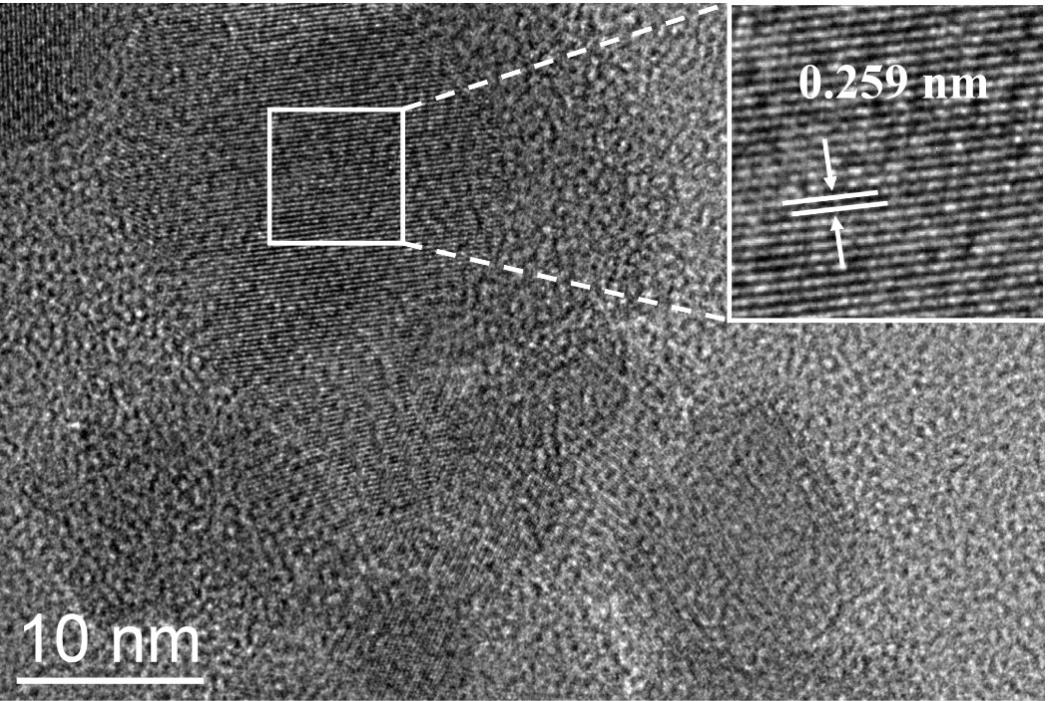
**

**Figure S3.** The MNPs electron diffraction image.

**
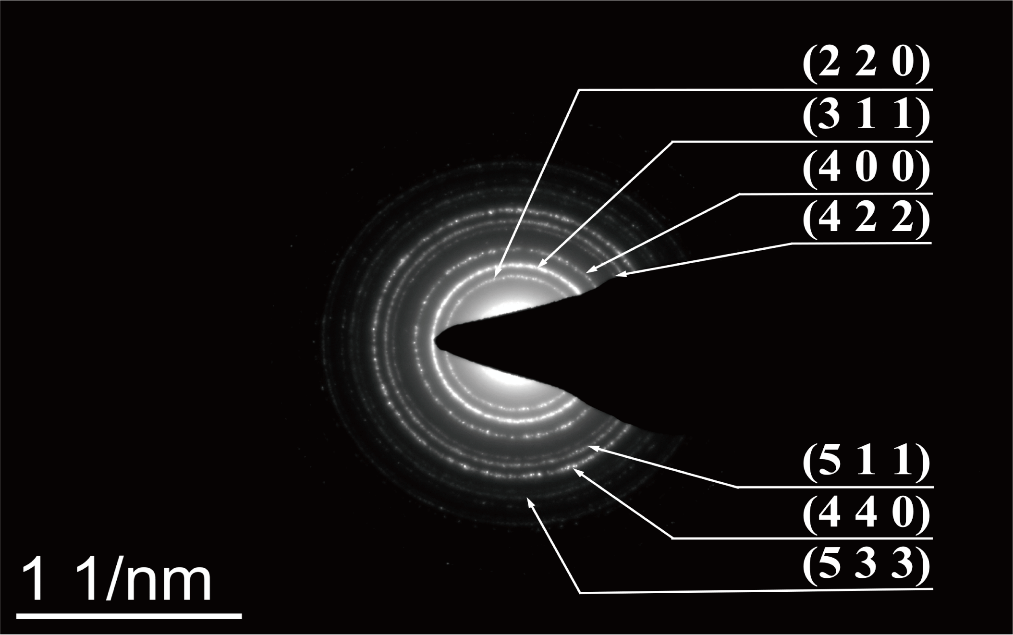
**

**Figure S4.** The X-ray diffraction spectra of MNPs.

**
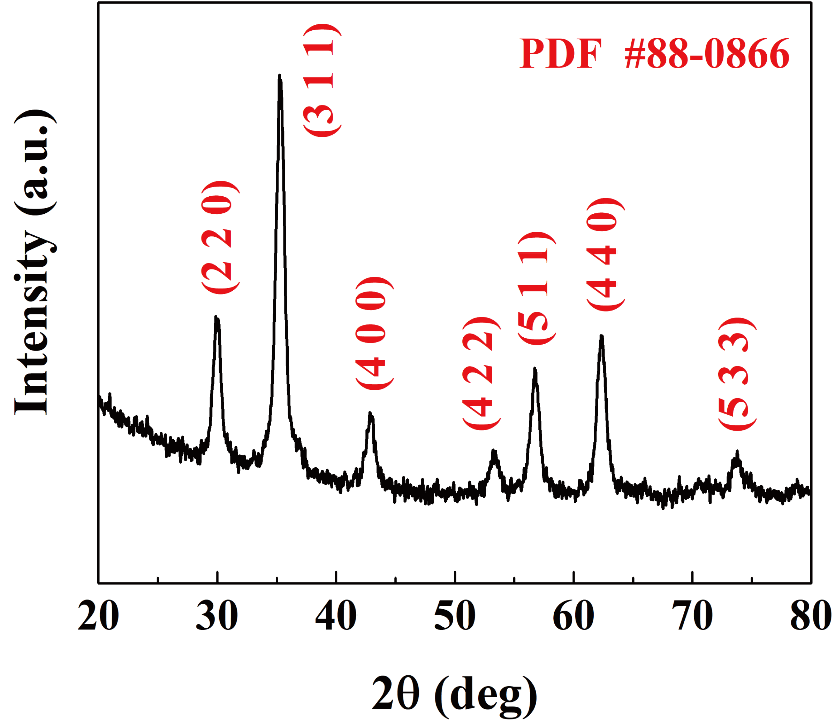
**

**Figure S5.** Zero-field-cooling and field-cooling (ZFC-FC) curves of MNPs

**
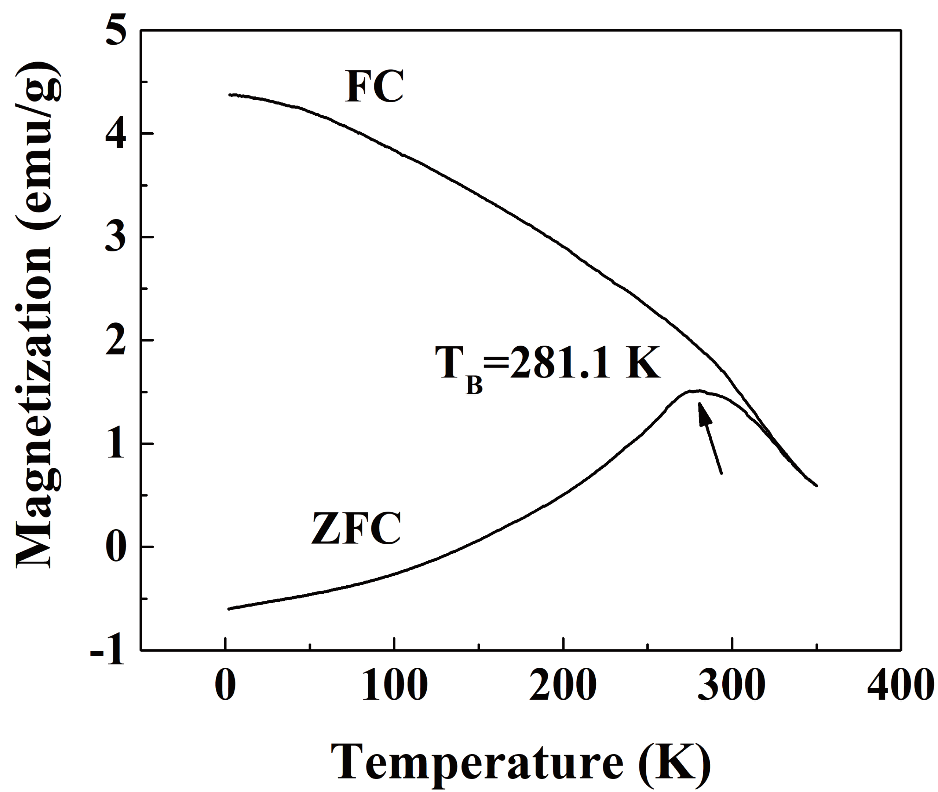
**

**Figure S6.** Thermogravimetric curve of MNPs, showing the change of the first derivative of the normalized mass-temperature curve against temperature.

**
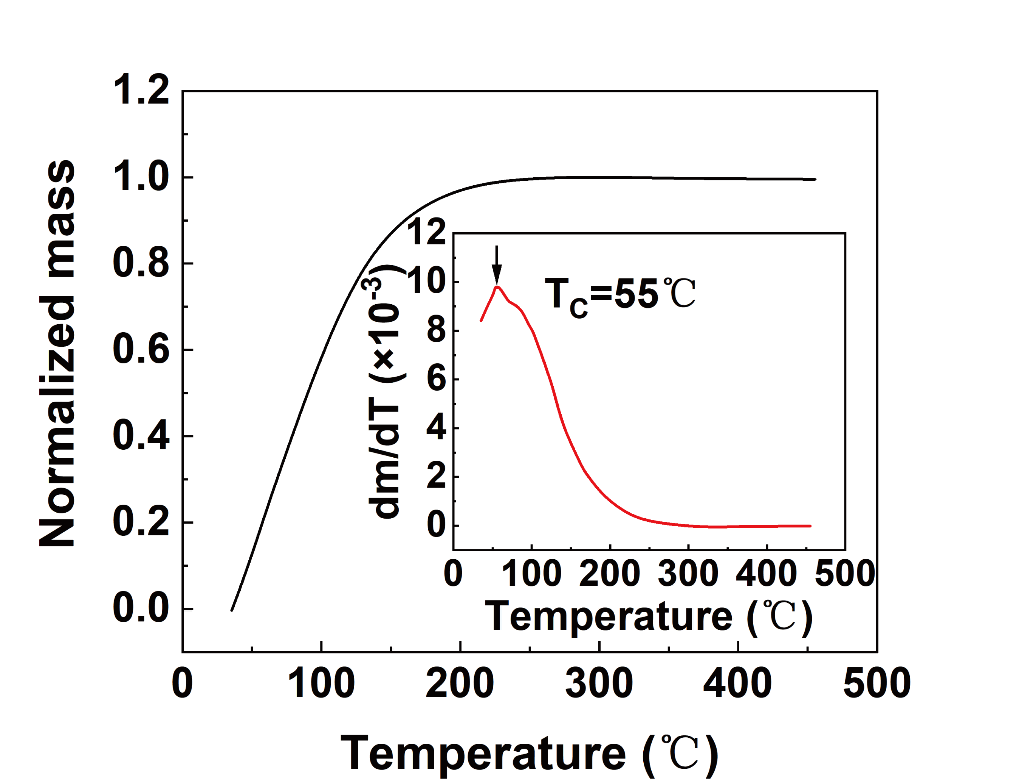
**

**Figure S7.** Diagram of composite seed.

**
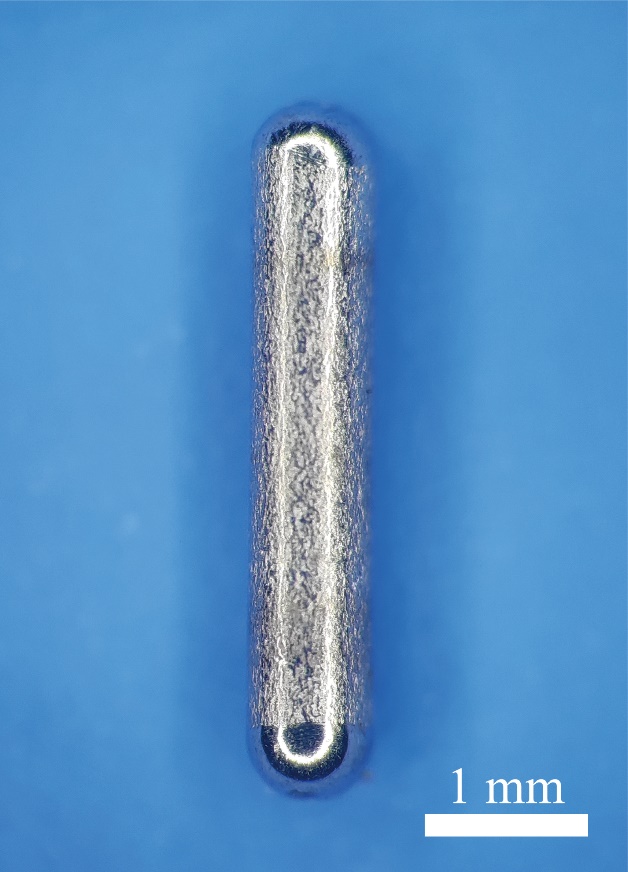
**

**Figure S8.** Temperature contour section figures of four composite seeds heating tumor in coronal plane, sagittal plane and transverse plane at different distance (D).

**
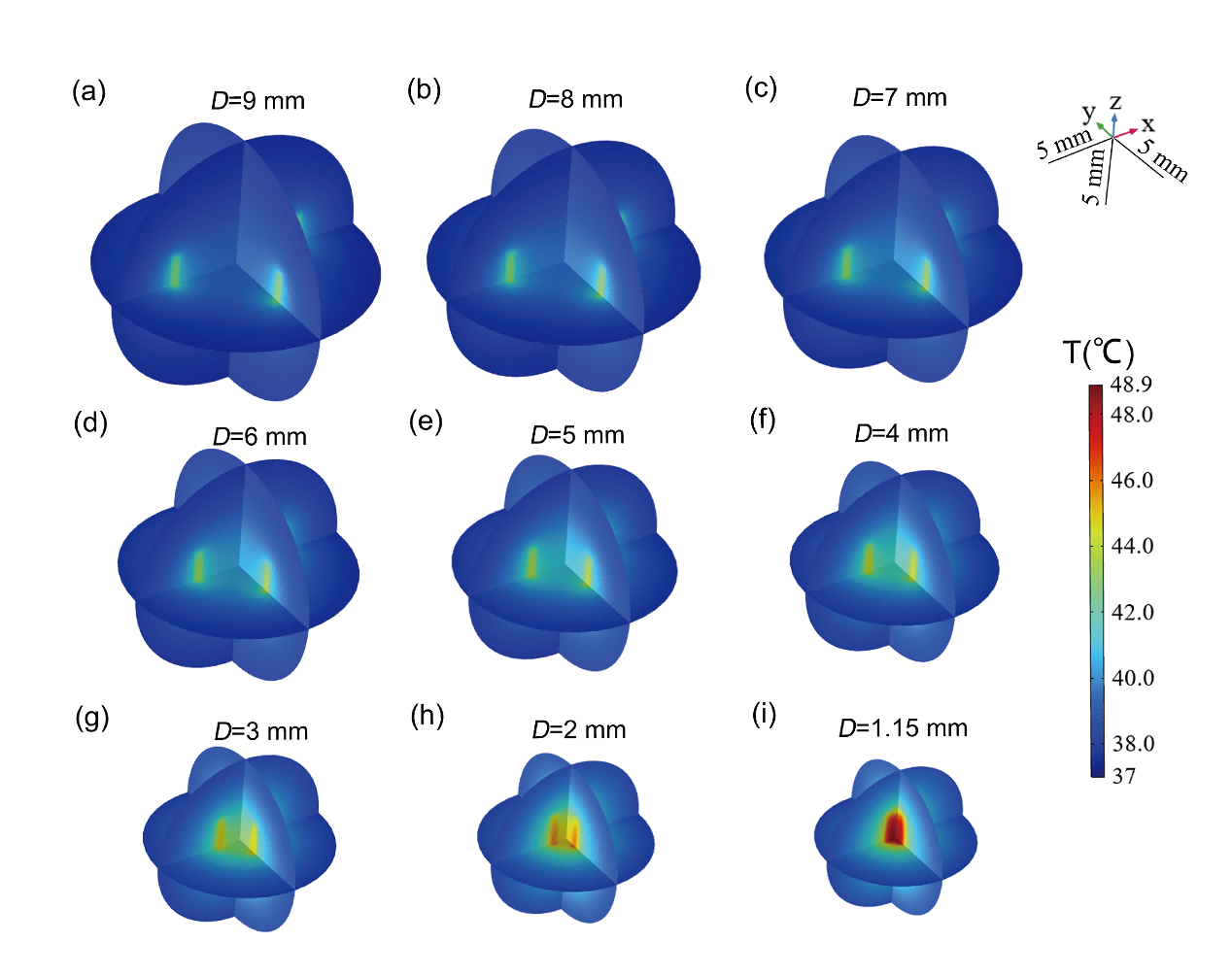
**

**Figure S9.** Temperature distributions in the tumor and seeds along central line of coronal plane of four seeds.

**
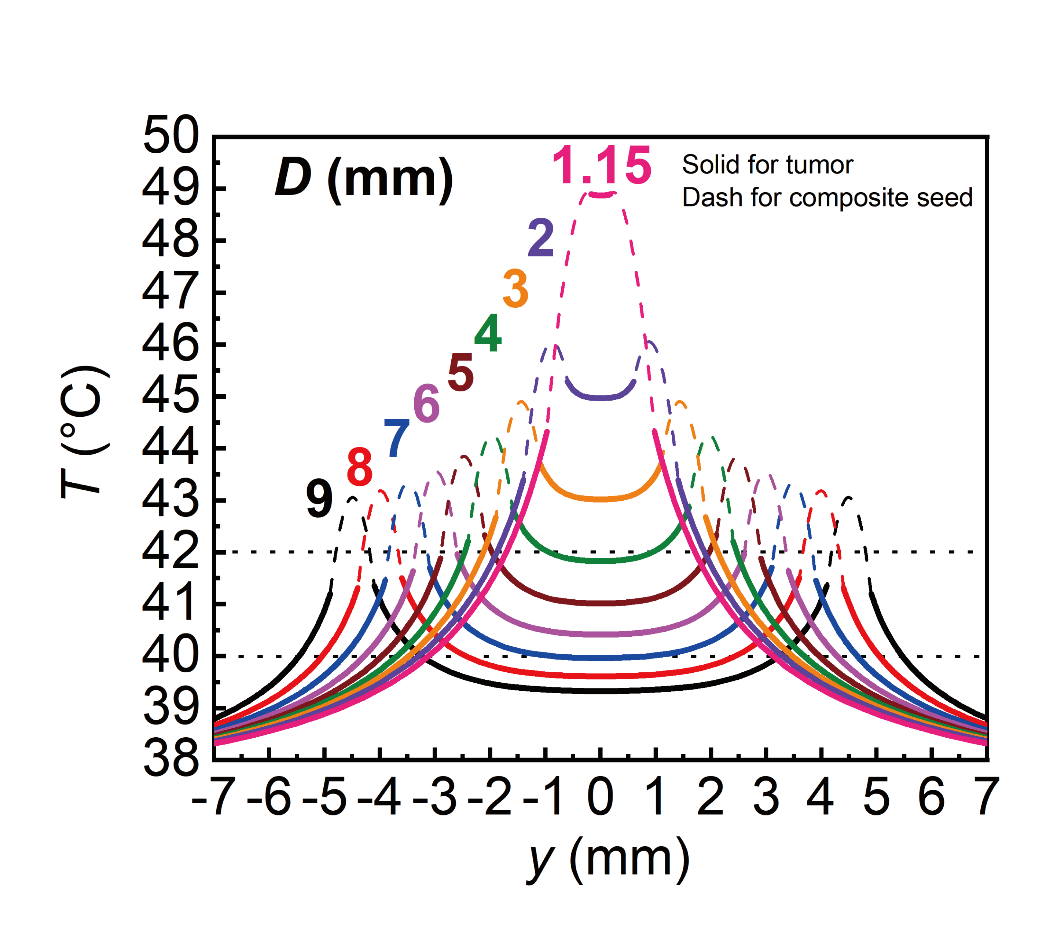
**

**Figure S10.** Effective therapeutic volume (Ve) and overheated volume (Vo) of tumor when four seeds heating at different distance (D). The contour figures are half volumes of corresponding tumor area.

**
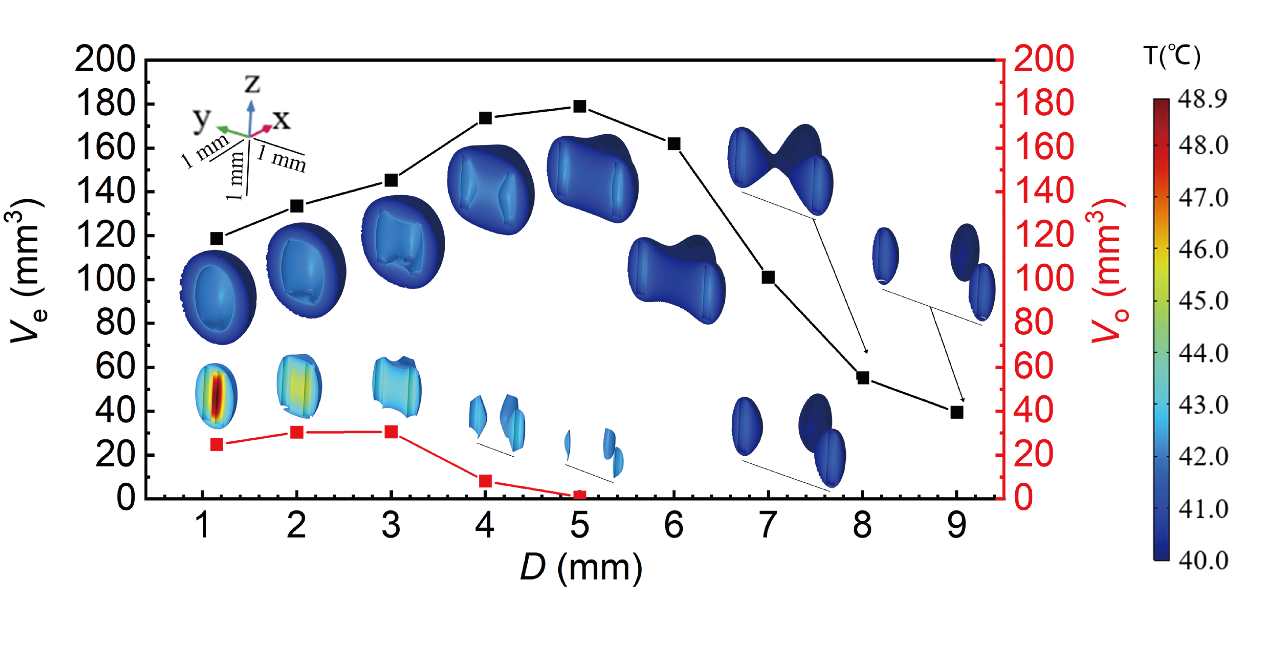
**

**Figure S11.** Hyperthermia simulation temperature contour figures of superficial tumor in: (a) coronal plane; (b) surface of tumor and healthy tissue.

**
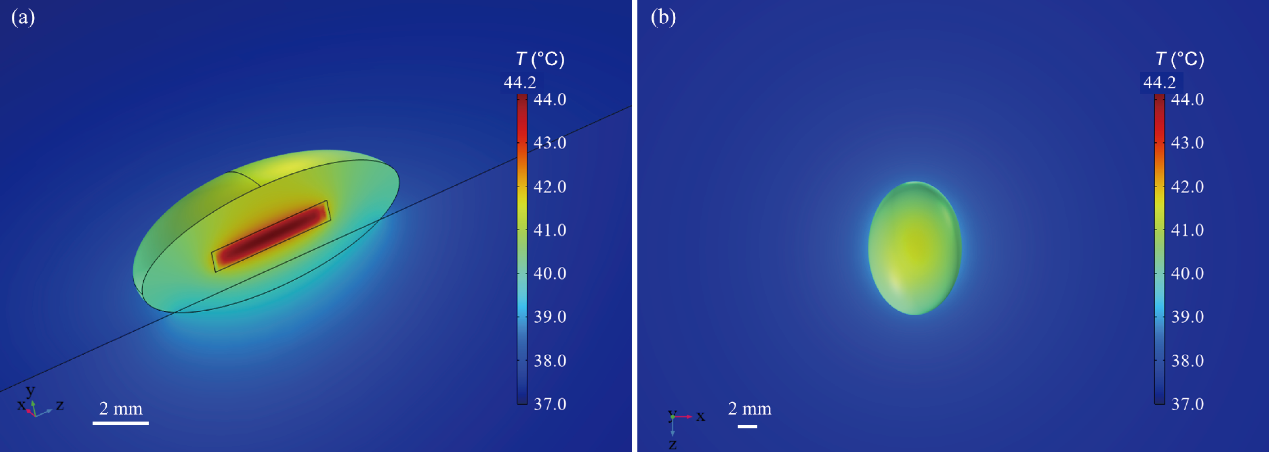
**

**Figure S12.** Tumor growth curves of three groups and changes in mouse body weight for Hepa 1-6 mouse subcutaneous tumor models.

**
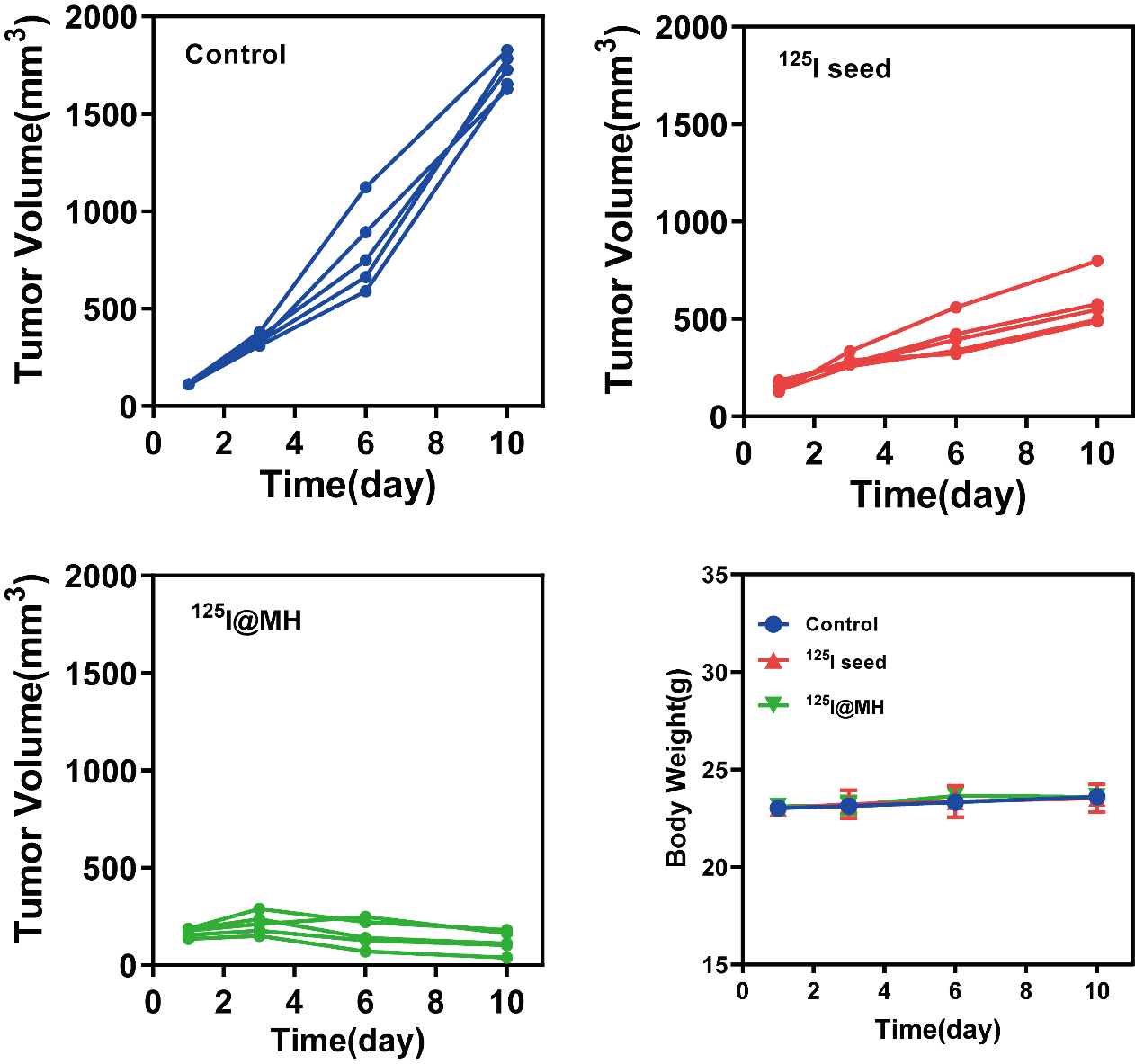
**

**Figure S13.** H&E, KI67 (brown), and TUNEL (green fluorescence) staining images of subcutaneous Hepa 1-6 tumor tissues in three groups of mice.

**
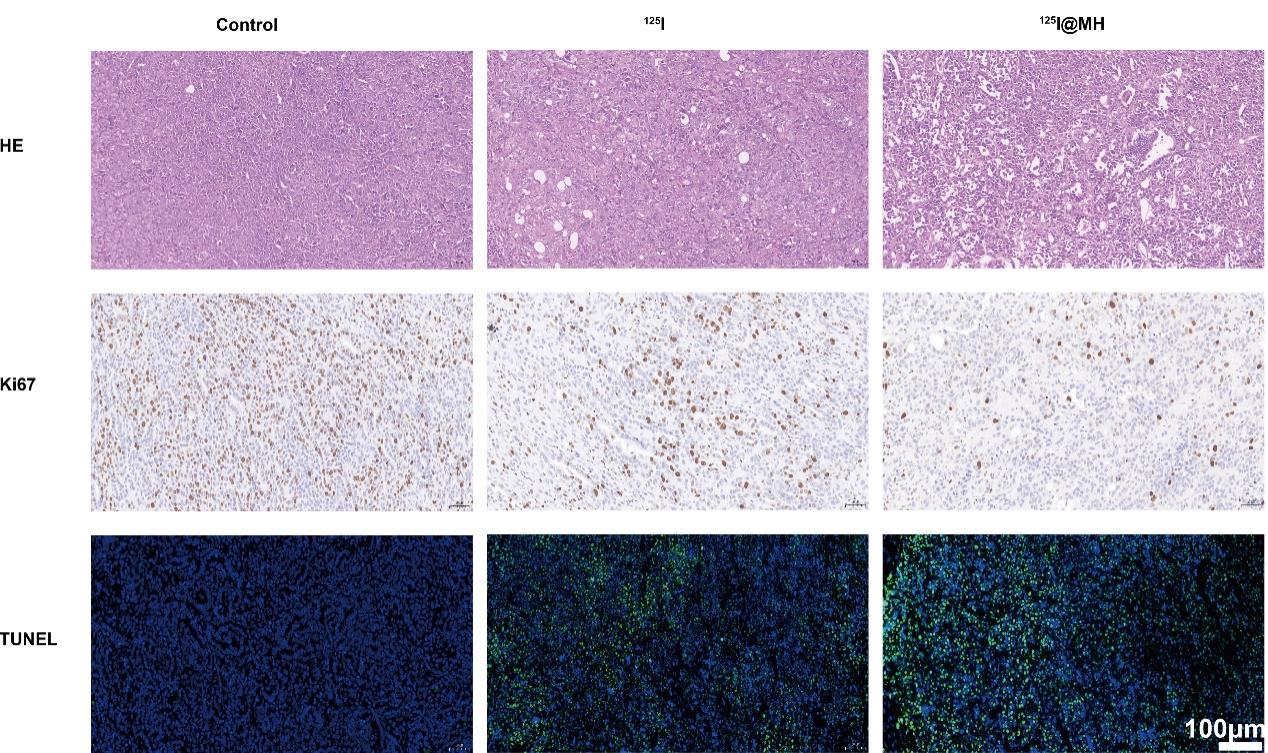
**

**Figure S14.** Biosafety evaluations include H&E staining images of the heart, liver, spleen, lungs, and kidneys in mice, as well as serum biochemical parameters (ALT, AST and creatinine), complete blood counts (WBC, RBC and hemoglobin) and weight growth curves tests.

**
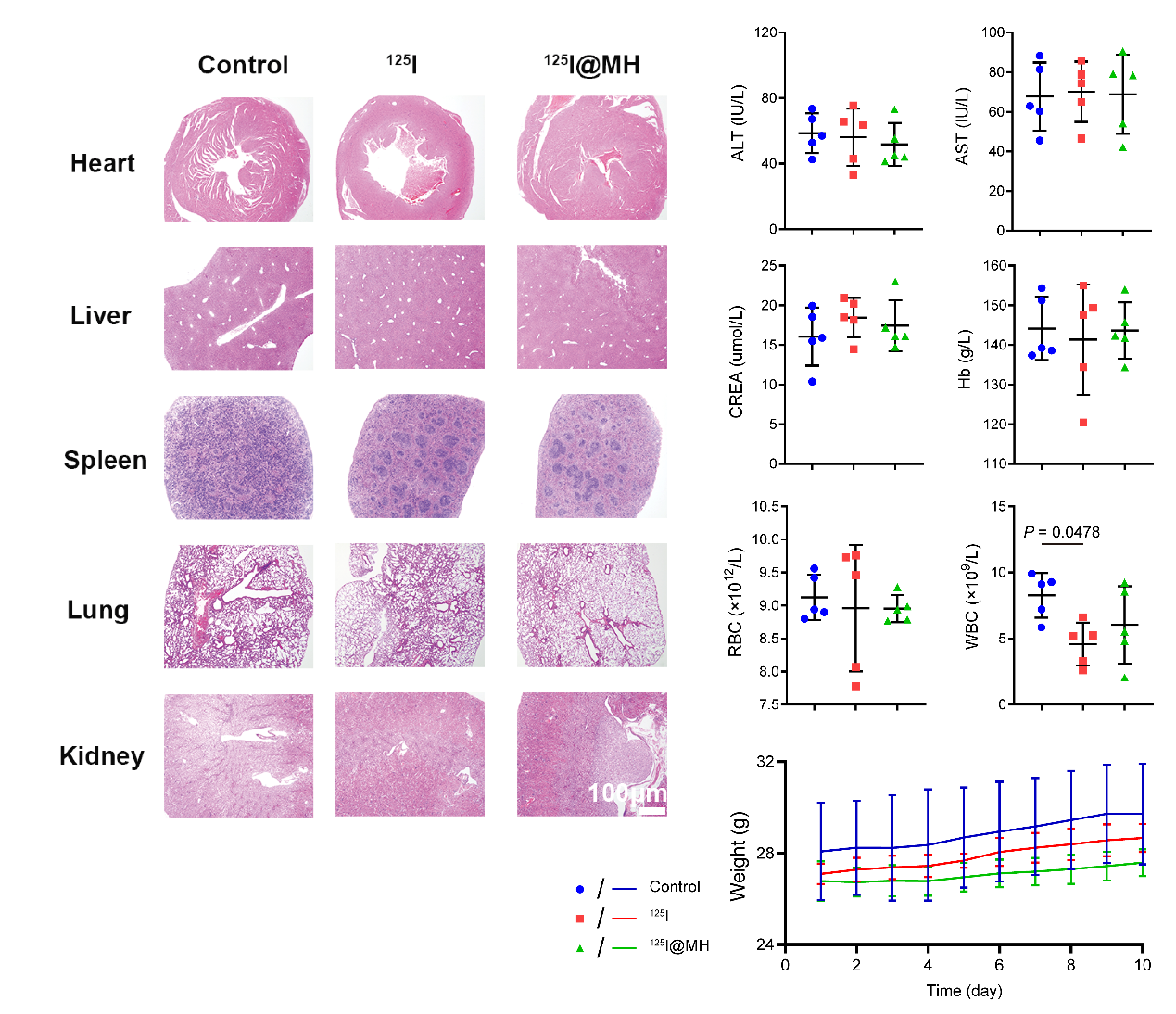
**

**Figure S15.** KI67 (brown) and TUNEL (green fluorescence) staining images of orthotopic N1S1 liver cancer tissues in three groups of rats.

**
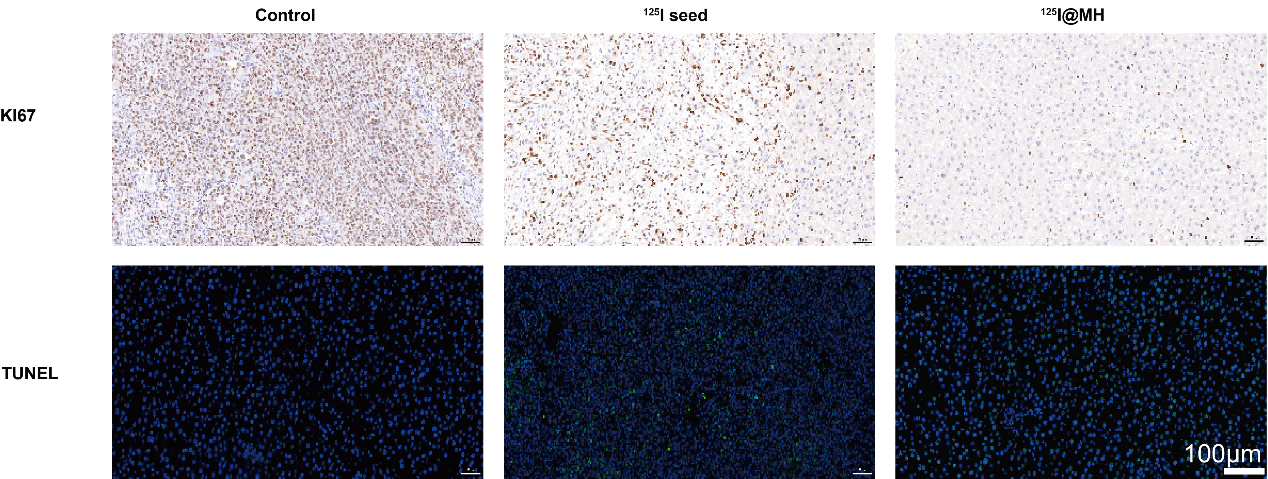
**

**Figure S16. ¹²⁵I@MH composite seed enhances the treatment efficiency of brachytherapy in prostate cancer.** (A-B) Harvested tumors and comparisons of tumor weights from RM-1 cell-treated subcutaneous tumor-bearing mice treated with different therapeutic methods. (F) Growth curves of RM-1 subcutaneous tumors in mice after different treatments.


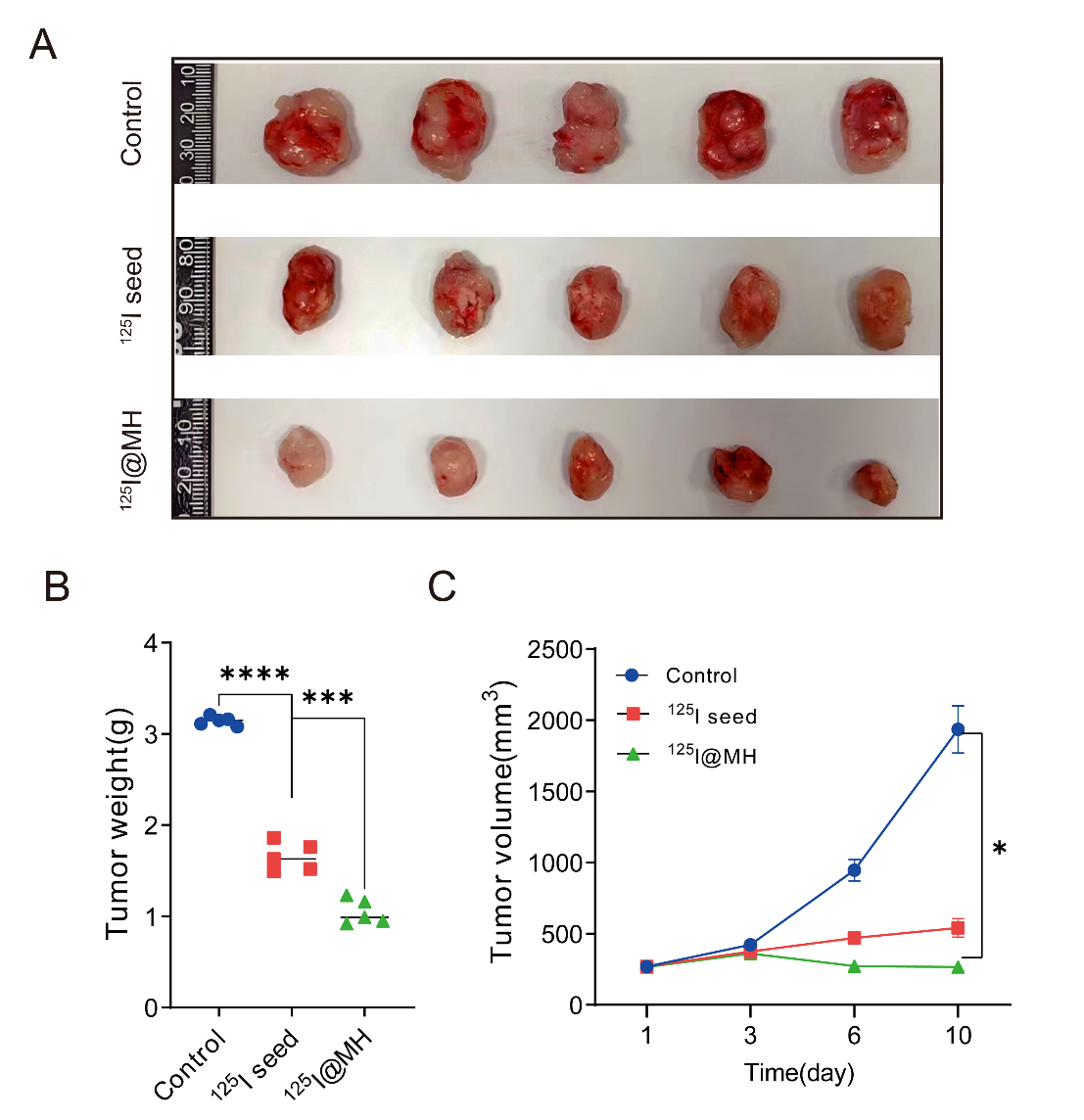


**Figure S17.** The detailed gating strategies for the flow cytometry of Cd274^+^ neutrophils.

**See Supporting Information – Fig S17.pdf**

**Figure S18.** The detailed gating strategies for the flow cytometry of Ifn-γ+ T cells.

**See Supporting Information – Fig S18.pdf**

**Figure S19.** The detailed gating strategies for the flow cytometry of Pd-1^+^ T cells.

**See Supporting Information – Fig S19.pdf**

**Figure S20.** The detailed gating strategies for the flow cytometry of Ccr2^+^ macrophages.

**See Supporting Information – Fig S20.pdf**

**Figure S21.** The detailed gating strategies for the flow cytometry of Cd206^+^ macrophages.

**See Supporting Information – Fig S21.pdf**

**Figure S22. Pan-cancer immune reconstruction triggered by 125I@MH therapy.** (A) UMAP plot of the single-cell landscape for subcutaneous tumor of PCa, colored by general cell type. (B) Stacked bar chart showing the proportion of general immune cell types across different treatment groups. (C) UMAP plot of the lymphocytes, colored by subtypes. (D) Stacked bar chart showing the proportion of lymphoid subtypes across different treatment groups. (E) UMAP plot of the phagocytes, colored by subtypes. (F) Stacked bar chart showing the proportion of phagocyte subtypes across different treatment groups. (G-H) Flow cytometry analysis of PCa subcutaneous tumors comparing the proportion of Pd-1+ T cells across different treatment groups.


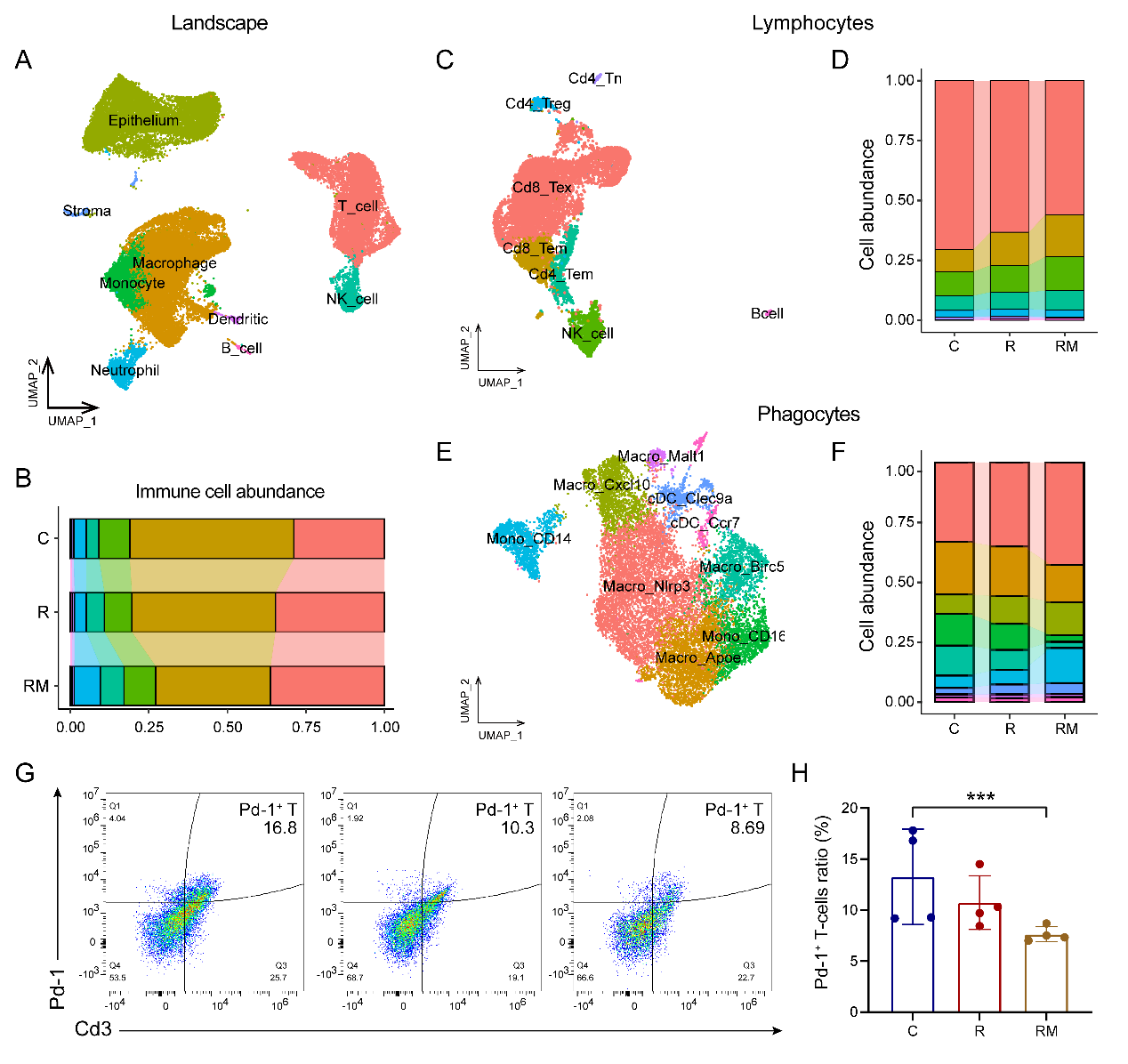


**Figure S23.** The detailed gating strategies for the flow cytometry of Pd-1^+^ T cells.

**See Supporting Information – Fig S23.pdf**

**Figure S24. The assembly of composite seed.**

**
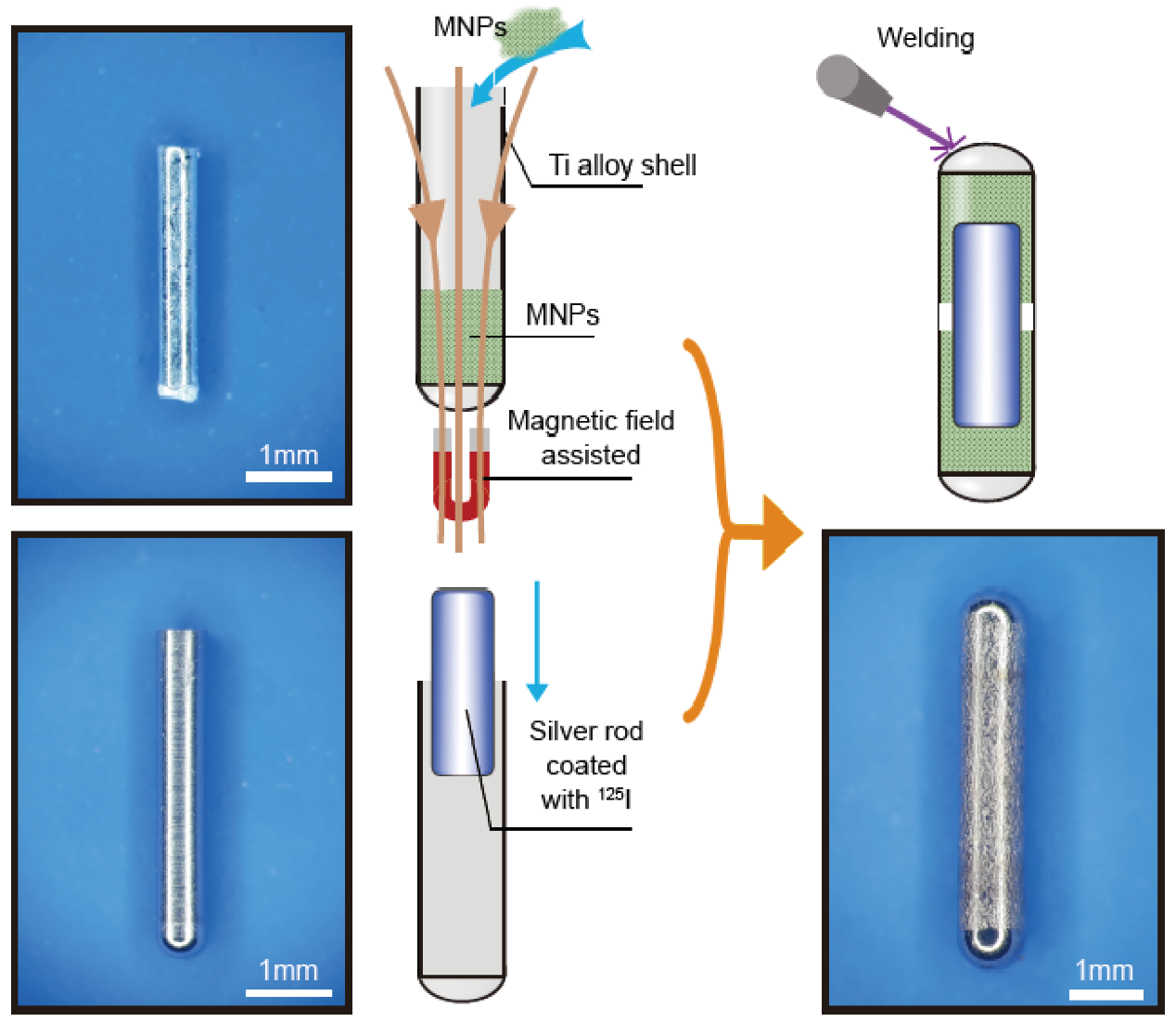
**

**Figure S25.** Hyperthermia simulation model of composite seeds arranged at the center, surrounded by tumor and healthy liver tissue. The following are diagrams for (a) single oseed, (b) two seeds; (c) four seeds; (d) different components of the seed; (e) different planes of the model.

**
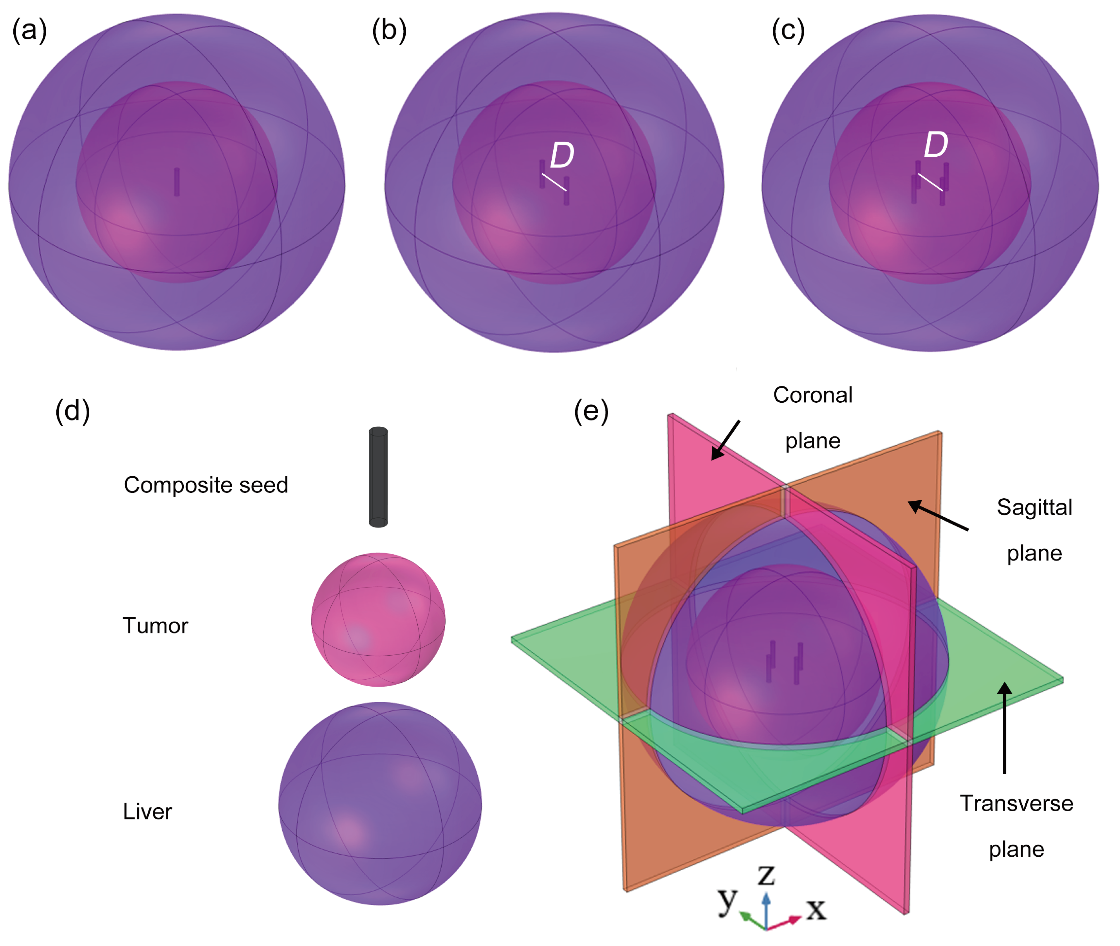
**

**Figure S26.** Hyperthermia simulation model of superficial tumor of *in vivo* experiment.

**
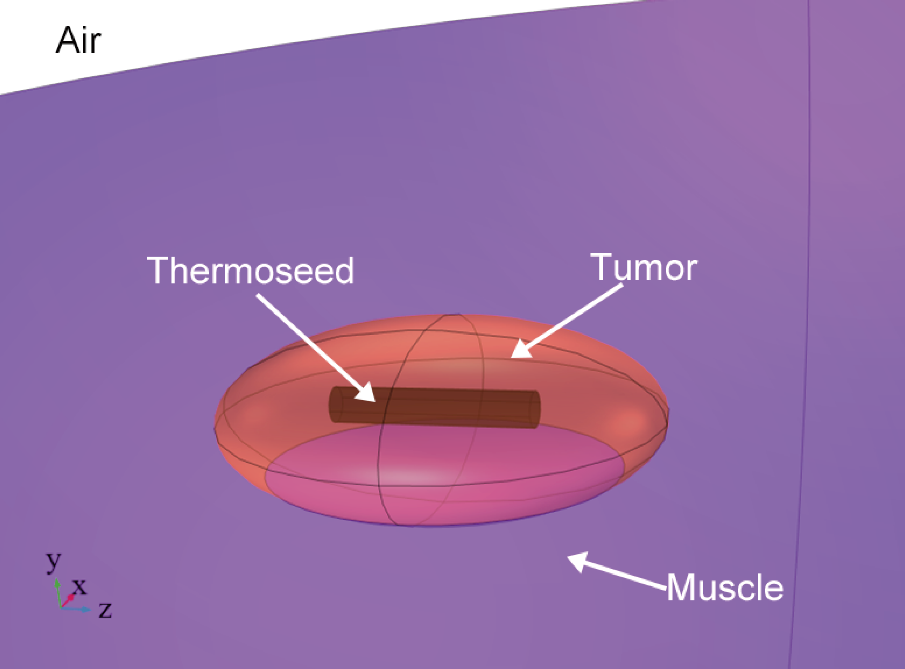
**
