# Supplementary material for: A Novel Self‐Regulated, Non‐Directional Magnetic Thermos‐Brachytherapy 125I Seed Enhances Anticancer Efficacy by Rescuing Immune Escape
Source: Adv Sci (Weinh). 2025 Aug 29;12(43):e08091. doi: 10.1002/advs.202508091 (PMC12631875; doi:10.1002/advs.202508091)

Figure S17. The detailed gating strategies for the flow cytometry of Cd274+ neutrophils.

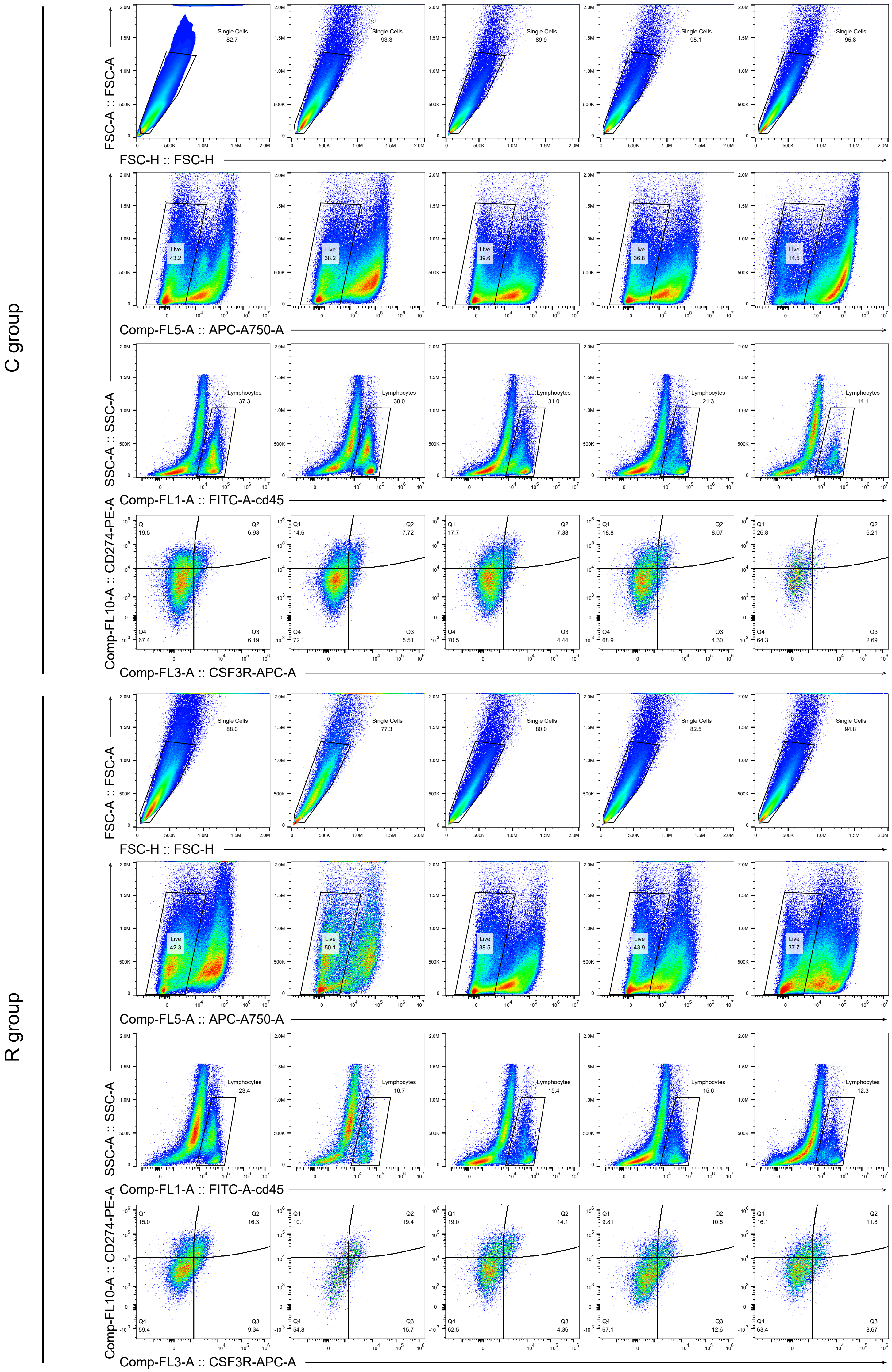

Supplement: Supplementary file 2 — Supporting Figures [file ADVS-12-e08091-s002.zip › advs71495-sup-0002-FigureS17.pdf]
